# Conformational dynamics linked to domain closure and substrate binding explain the ERAP1 allosteric regulation mechanism

**DOI:** 10.1038/s41467-021-25564-w

**Published:** 2021-09-06

**Authors:** Zachary Maben, Richa Arya, Dimitris Georgiadis, Efstratios Stratikos, Lawrence J. Stern

**Affiliations:** 1 Department of Pathology, University of Massachusetts Medical School, Worcester, MA USA; 2 Department of Chemistry, National and Kapodistrian University of Athens, Athens, Greece

**Keywords:** Enzyme mechanisms, SAXS, X-ray crystallography

## Abstract

The endoplasmic-reticulum aminopeptidase ERAP1 processes antigenic peptides for loading on MHC-I proteins and recognition by CD8 T cells as they survey the body for infection and malignancy. Crystal structures have revealed ERAP1 in either open or closed conformations, but whether these occur in solution and are involved in catalysis is not clear. Here, we assess ERAP1 conformational states in solution in the presence of substrates, allosteric activators, and inhibitors by small-angle X-ray scattering. We also characterize changes in protein conformation by X-ray crystallography, and we localize alternate C-terminal binding sites by chemical crosslinking. Structural and enzymatic data suggest that the structural reconfigurations of ERAP1 active site are physically linked to domain closure and are promoted by binding of long peptide substrates. These results clarify steps required for ERAP1 catalysis, demonstrate the importance of conformational dynamics within the catalytic cycle, and provide a mechanism for the observed allosteric regulation and Lys/Arg528 polymorphism disease association.

## Abstract (web summary)

The endoplasmic-reticulum aminopeptidase ERAP1 processes peptides for antigen presentation. Here, the authors assess ERAP1 conformational states in solution, providing insight into the molecular mechanisms of ERAP1 substrate-length dependent catalytic activity and regulation, including the effects of autoimmune disease-associated polymorphism.

## Introduction

Endoplasmic-reticulum aminopeptidase 1 (ERAP1) is a key determinant of antigen processing and presentation. In this role, ERAP1 activity digests peptides in class I major histocompatibility complex (MHC-I) antigen-processing pathway, in a manner that can create or destroy peptide epitopes for presentation to CD8 T cells^1^. Polymorphisms within ERAP1 correlate with predisposition to autoimmune diseases, pre-eclampsia, and cancer^2,3^. The ERAP1 single-nucleotide polymorphism (SNP) rs30187, which encodes lysine or arginine at position 528, has been associated with autoimmune diseases in epistasis with specific MHC alleles^4,5^ and alters the set of peptides presented by MHC^6,7^. In biochemical assays, lysine/arginine 528 substitution affects ERAP1’s peptide hydrolysis activity^8^. How this polymorphism 25 Å away from the active site zinc affects enzymatic activity and specificity is unknown.

ERAP1 is a member of the M1 aminopeptidase family, which is found in all domains of life and plays numerous functional roles^9^. These proteins contain a thermolysin-like domain^10^ (domain II), which holds the active site. The M1 aminopeptidase family has a conserved HEAT-motif-repeat domain (domain IV) forming a bowl-shaped C-terminal structure^9^. In most crystal structures of this family, domain IV is closely associated with domain II and forms an internal pocket that separates the active site from the bulk solvent. This interdomain interaction sterically blocks substrate binding and product release, and it was hypothesized that aminopeptidases in this family must change structure during the catalytic cycle in order to accommodate these steps^9^.

The first crystal structures of ERAP1 provided some structural underpinning to this picture. One crystal structure matched the closed conformation that had been observed for other family members^11^. Two other crystal structures captured an open conformation, in which domain IV had undergone a rigid-body translocation away from domain II, exposing the internal cavity to bulk solvent^11,12^. Along with the domain IV reorientation, the active site had reorganized with tyrosine 438 rotating away in a position not optimal for catalysis. This suggested a connection between the conformational state and catalytic activity. A similar motion of the homologous catalytic tyrosine 549 was later observed in crystal structures of insulin-regulated aminopeptidase (IRAP), in this case linked to the presence of a transition-state analog in the catalytic center^13–15^.

ERAP1 has an unusual substrate-length dependence, efficiently trimming peptides 10–15 residues long but sparing shorter peptides^16,17^, well suited to its role in processing peptides for loading onto MHC-I proteins, which typically bind peptides of ~9 residues. A connection between the active site and a hypothesized distal regulatory site within ERAP1 has been suggested previously to explain ERAP1 substrate-length dependence and allosteric activation^12^. ERAP1 hydrolysis of activated amino-acid substrates such as leucine-*p*-nitroanilide (Leu-pNA) shows deviations from Michaelis–Menten behavior suggestive of cooperativity within the catalytically active monomer^12^. In addition, catalysis of short substrates is increased by the presence of short peptides 4–14 residues in length^12,18^. These data were interpreted in terms of a mechanism whereby the allosteric modulators bind within the overall substrate-binding cavity but distal to the active site, inducing conversion to a more active form^12^. These findings, along with data showing preferential hydrolysis of substrates longer than eight amino acids^16^, support a model that an optimal length substrate peptide simultaneously binds the active site and a distal regulatory site that activates catalysis^12,17^. Binding sites within the overall substrate-binding cavity have been identified for two small-molecule ERAP1 allosteric modulators^19,20^, one of which overlaps with the binding site identified for the C-terminus of peptides with a hydrophobic terminal residue^21,22^, but how occupancy of these sites induces activation, and whether activation is related to domain reorganization, remains unknown.

Here, we examine conformational states of ERAP1 using small-angle X-ray scattering (SAXS) and observe that ERAP1 adopts a largely open conformation in solution, but closes in the presence of substrates, inhibitors, or an allosteric activator that binds far from the active site at an interdomain junction. The disease-related polymorphic position 528 (lysine/arginine) alters the stability of the closed conformation and modulates an interdomain electrostatic interaction with glutamate 913, providing a mechanistic explanation for altered catalytic efficiency. The crystal structure of a substrate-mimic inhibitor bound to ERAP1 in the open conformation shows how helix 4a folds in the presence of substrate, coupling active-site alterations to large-scale domain motions. The binding site for peptide substrate C-terminal hydrophobic amino-acid residues identified previously in crystal structures^21,22^, along with alternate sites identified here by chemical cross-linking, are used to model the effect of peptide binding on domain closure energetics. These results suggest a model for regulation of ERAP1 activity that connects substrate binding and active-site reorganization through a large-scale domain closure motion promoted by binding long peptide substrates.

## Results

### ERAP1 adopts an open conformation in solution and closes with active site occupancy

ERAP1 has been observed to adopt open and closed conformations in X-ray crystal structures^11,12^, but whether these states interconvert in solution, and the relevance of the conformational change to catalysis, has not been determined. In order to investigate conformational transitions of ERAP1 in solution, we used small-angle X-ray scattering (SAXS). In initial experiments, we used *ERAP1* allele IV isoform 2 (Supplementary Fig. S1a), as previously used for structural characterization^12^, in the presence or absence of the broad-range aminopeptidase inhibitors bestatin^23^ or leucinethiol (LeuSH)^24^, which target the substrate S1 and S1’ subsites and active site zinc, respectively^11,12,23–25^. In the presence of either of these inhibitors, ERAP1 becomes more compact, with the radius of gyration, *R*_g_, decreasing by ~2 Å (Supplementary Fig. S2a). To investigate the effect of a more physiological ligand on ERAP1 conformation, we used the octamer peptide SIINFEKL. This peptide is generated efficiently from longer substrates by ERAP1 in vitro^17^ and in vivo^26^, and itself is a suboptimal ERAP1 substrate^16^. In the presence of a saturating amount of SIINFEKL, ERAP1 adopted a closed conformation (Supplementary Fig. S2a).

**Fig. 1:**
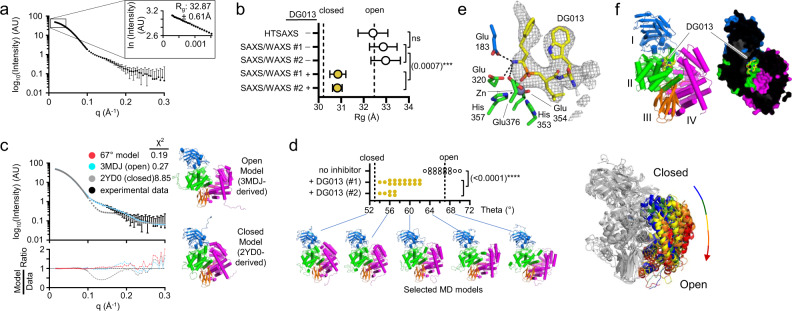
ERAP1 adopts an open conformation in solution and closes when bound to the substrate and substrate-mimic inhibitors. **a** Representative ERAP1 SAXS curve collected using SAXS/WAXS data collection. Data are for ERAP1 reference sequence (isoform I) in the absence of inhibitor or substrate (*n* = 1 sample was assessed in an independent experiment). Error bars represent uncertainty in log intensity values from radial averaging of scattering profiles. Inset, Guinier plot showing *R*_g_ determination using a linear fit of low-resolution scattering data (*n* = 1, *q* < 0.04 Å^−1^); fit value for slope and associated uncertainty are shown. **b** DG013 induces closed ERAP1 conformation as measured by *R*_g_. Each data point represents an independent experiment (*n* = 1) processed individually, with the error of linear fit for each dataset shown as error bars. Model for open and closed conformations are based on previously determined ERAP1 crystal structures (2YD0 and 3MDJ chain B) shown for reference are right, with calculated *R*_g_ values indicated with dotted vertical lines. An unpaired two-tailed *t* test was used to assess the significance of the difference between SAXS/WAXS *R*_g_ values for ERAP1 in the presence or absence of DG013 from two independent experiments. **c** ERAP1 experimental SAXS curve (*n* = 1) from panel **a** overlaid with calculated SAXS curves generated from three structural models. A largely open model derived from molecular dynamics simulation with *q* = 67° fits best. Fit residuals for these three models are shown below. **d** DG013 induces closed ERAP1 conformation as measured by SAXS curve fitting to structural models from molecular dynamics. Models that fit experimental SAXS data with *χ*^2^ < 1 are shown as points on the graph from an independent experiment, forming a distribution of models that each fit scattering data within experimental uncertainty. An unpaired two-tailed *t* test was used to assess significance. Selected ERAP1 models from MD simulation that demonstrate the relationship between angle theta and structural conformation are shown below and on the right is an overlay of selected MD models showing the hinge-like motion of domain IV. **e** DG013 binds to ERAP1 active site. Polder map (gray mesh) contoured at 3.5 σ shows DG013 electron density at ERAP1 active site. **f** DG013-bound ERAP1 crystal structure is in the closed conformation. Shown are cartoon and surface representations colored by domain as indicated. Surface cutaway reveals DG013 bound at the active site. Source data are provided as a Source Data file.

To facilitate detailed SAXS analysis, we used a different ERAP1 construct (allele II isoform 1, Supplementary Fig. S1), which lacks a nine-residue unstructured C-terminal tail and recombinant *myc* tag not visualized in the X-ray structure. Removing these unstructured regions simplified fitting the experimental scattering data to molecular models. We collected solution X-ray scattering data including wide-angle WAXS data to allow for direct measurement of the water scattering profile (maximum intensity at *q* = 2 Å^−1^), producing high-quality background-subtracted scattering curves^27^. *R*_g_ values as determined by Guiner fitting matched the *R*_g_ value calculated for the open conformer observed by X-ray crystallography, as previously reported^28^ (Fig. 1a). We observed no differences in *R*_g_ for samples of this isoform collected by either conventional SAXS or SAXS/WAXS (Fig. 1b, open circles). The SAXS/WAXS buffer subtraction method improves data quality at higher *q* values, allowing higher-resolution analysis in which the full range of scattering data can be fit to experimental or computational atomic models. The full-profile dataset fit better to a model based on an open-crystal structure (Fig. 1c, cyan symbols) than to one based on the closed crystal structure (Fig. 1c, gray symbols). For the open-crystal structure model, residuals for the fit of experimental to calculated scattering intensities were small and evenly distributed throughout the resolution range (Fig. 1c, bottom pane), and were smaller than expected experimental uncertainties based on radial averaging of observed scattering patterns (Fig. 1c, black bars). To explore other conformers, we used a set of 45 structural models selected from a molecular dynamics simulation^28^ that sample a range of opening angles with opening angle theta^28^ ranging from 54 to 74° (Fig. 1d). Of these, the best fitting model had theta = 67° (Fig. 1c, red symbols), similar to that observed for the open-crystal structure, but with a lower *Χ*^2^ value. To account for uncertainties in the experimental data, we considered all models with *Χ*^2^ < 1. This provided a cluster of models fitting the experimental ERAP1 data, with theta angles bracketing the value calculated for the open-crystal structure (Fig. 1d, open symbols).

To examine the effect of active-site occupancy on ERAP1 conformation in solution, we used a tight-binding tripeptide analog DG013 (IC_50_ = 33 nM^29^), which has a phosphinic group expected to mimic the tetrahedral intermediate that would form at the scissile peptide bond during catalysis (Supplementary Fig. S1c). We determined the X-ray crystal structure of ERAP1 bound to DG013 (Table 1, Supplementary Table 1, Supplementary Figs. 3 and 4, see “Methods” for details) and confirmed that DG013 binds as expected to the ERAP1 active site (Fig. 1e, f and Supplementary Table S1a), with the enzyme adopting the closed conformation in the crystal (Fig. 1f and Supplementary Fig. S3a–f). The binding pose is similar to that of another phosphinic peptide, DG046, bound to ERAP1^30^, but distinct from DG013 bound to ERAP2, most likely due to lack of an aromatic P2’-binding site.

**Table 1:** Data collection and refinement statistics for ERAP1 bound to phosphinic inhibitors.

	ERAP1 in complex with phosphinic pseudotripeptide inhibitor DG013 (PDB ID 6M8P)	ERAP1 in complex with phosphinic pseudodecapeptide inhibitor DG014 (PDB ID 6MGQ)
Wavelength (Ã)	0.979	1.110
Resolution range (last shell)	41.7–3.3 (3.4–3.3)	56.1–2.9 (3.0–2.9)
Space group	P2 21 21	P1 21 1
Unit cell a, b, c	125.79, 548.68, 589.06	56.14, 234.7, 132.28
α, β, γ	90, 90, 90	90, 93.12, 90
Total reflections	6,551,362 (1,046,163)	734,382 (80,646)
Unique reflections	601,903 (19,094)	73,472 (1842)
Multiplicity	10.9 (10.5)	10.0 (8.8)
Completeness (%)	74.7 (19.2)	81.7 (20.1)
Completeness, elliptical (%)	87.6 (51.9)	99.2 (98.9)
Mean I/sigma (I)	2.68 (0.54)	20.11 (0.56)
Wilson B-factor	29.6	59.5
R-merge	1.121 (4.858)	0.657 (4.604)
R-meas	1.177 (5.105)	0.692 (4.896)
R-pim	0.353 (1.55)	0.215 (1.631)
CC1/2	0.952 (0.145)	0.861 (0.234)
CC*	0.988 (0.503)	0.962 (0.616)
Reflections used in refinement	449,952 (19,086)	60,060 (1841)
Reflections used for R-free	2123 (83)	2987 (92)
R-work	0.285 (0.330)	0.198 (0.309)
R-free	0.295 (0.361)	0.256 (0.371)
CC (work)	0.725 (0.426)	0.865 (0.564)
CC (free)	0.758 (0.280)	0.844 (0.448)
Protein molecules per ASU	22	3
Number of non-hydrogen atoms	155,927	20,671
Number of macromolecules	151,544	20,279
Number of ligands	4383	138
Number of solvent	NA	39
Protein residues	18,942	2562
RMS (bonds)	0.004	0.005
RMS (angles)	0.64	0.79
Ramachandran favored (%)	95.94	94.13
Ramachandran allowed (%)	4.06	5.52
Ramachandran outliers (%)	0	0.35
Rotamer outliers (%)	0.42	1.7
Clashscore	6.27	27.32
Average B-factor	39.5	70.7
Average macromolecules	39.0	70.7
Average ligands	55.8	59.0
Average solvent	NA	32.77
Number of TLS groups	1	14

In the presence of DG013, ERAP1 *R*_g_ decreases by ~2 Å, an effect observed in multiple measurements and for two independent protein preparations (Fig. 1b, yellow circles). Comparison of this experimental finding with *R*_g_ calculated for structural models derived from open and closed crystal structures (PDB ID 3MDJ and 2YD0, respectively, dotted vertical lines in Fig. 1b) shows that the ~2 Å *R*_g_ shift upon ERAP1 binding DG013 is consistent with a shift of the conformational equilibrium from open toward closed. Fitting the ERAP1 + DG013 scattering profiles to molecular dynamics structures revealed a cluster of models consistent with the experimental data (*Χ*^2^ < 1) with theta between 54 and 62° (Fig. 1d, yellow symbols). This cluster is distinct and nonoverlapping with that observed for ERAP1 in the absence of DG013, and consistent with a conformation similar to but slightly more open than observed for ERAP1 in closed-conformation crystal structures.

Overall, the SAXS analysis shows that ERAP1 adopts an open conformation in solution and closes upon binding substrate or substrate-like inhibitors.

### The crystal structure of a transition-state peptide analog bound to the open conformer reveals conformational changes induced by substrate binding

**Fig. 2:**
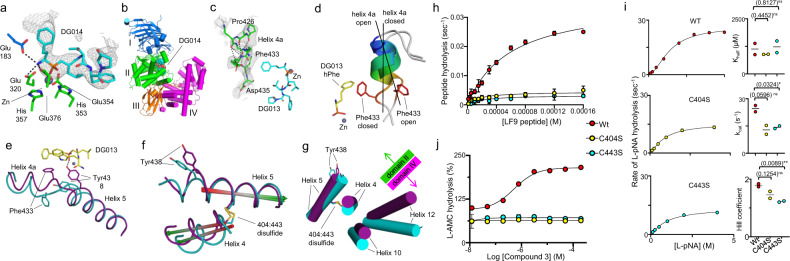
Ordering and reorientation of helix 4a near the active site with substrate binding and role of Cys403-443 disulfide in coupling active-site rearrangements with interdomain interactions. **a** DG014, cyan, is shown bound to ERAP1 active site. Polder map (gray mesh) contoured at 3.5 σ shows DG014 density in chain A. **b** ERAP1 was crystallized in the open conformation with DG014. Domains I (blue), II (green), III (orange), and IV (magenta) are shown as cartoons. DG014 is shown as cyan spheres. **c** Helix 4a is ordered along the bound inhibitor. Polder map (gray mesh) contoured at 2.8 σ. Asp435, a5 the C-terminal end of helix H4a, contacts DG014 at the P2’ and P4’ positions. **d** Helix H4a tilts and rotates between open and closed conformations. Helix 4a is colored as N- to C-terminal blue-red rainbow. Helix 4a tilts 35° between open and closed conformations (angle between helix central axes, black lines). Rotation along the helix central axis is depicted using Phe433 side chain for reference. DG013 homophenylalanine-phosphinyl group is shown bound at active site zinc. Phe433 in closed-conformation contacts S1’ homophenylalanine (hPhe) of DG013 in a T-shaped π–π interaction. **e** Phe433 motion occurs in conjunction with Tyr438 repositioning. Helix 4a moves as helix 5 twists, positioning Tyr438 for catalysis. Interaction with DG013 shown as dashed line. **f** Helix 5 rotation aligns Tyr438 for catalysis^11^. Helix 5 twist is coupled to reorientation of helix 4 through the 404:443 disulfide. **g** Helices 10 and 12 from domain 4 are adjacent to helix 4, coupling the domain interface to the substrate-binding site. **h** Mutation of residues participating in the Cys404-Cys443 disulfide bond by replacement with serine reduces LF9 peptide hydrolysis and (**i**) L-pNA enzymatic activity. The data are fit into Michaelis–Menten equation for peptide substrate (**h**) and allosteric sigmoidal for L-pNA with fit values for *K*_half_, *k*_cat_, and Hill coefficient are shown in (**i**). An unpaired two-tailed *t* test was used to assess the significance of the difference between *K*_half_, *k*_cat_, and hill coefficient for ERAP1 and Cys mutants from two independent experiments (*n* = 3). **j** Cys404-Ser and Cys443-Ser mutations block allosteric activation by compound **3**. Activity assays in (**h**, **j**) were performed in three independent experiments with two replicates (*n* = 2) for (**h**) and (**j**) in each independent assay. Error bars on (**h**–**j**) represent the variation between the samples in an independent experiment. Source data are provided as a Source Data file.

To begin to address how domain closure is coupled to catalysis, and to dissect structural features related to substrate binding from those related to the open-closed transition, we crystallized ERAP1 bound to the decamer phosphinic inhibitor, DG014, and determined the three-dimensional structure by X-ray crystallography (Table 1, Supplementary Fig. S3g–j, and Supplementary Table S1b) DG014 shares DG013’s phosphinic group and N-terminal residues but is seven amino-acid residues longer, and similarly inhibits ERAP1 with high potency (Supplementary Fig. S1c, S5). Like DG013, DG014 induced closing as assessed by SAXS/WAXS *R*_g_ and theta analysis (Supplementary Fig. S2). Serendipitously, we were able to obtain crystals of the ERAP1–DG014 complex in the open conformer, and we determined its structure by X-ray crystallography (Table 1, Supplementary Table 1, see “Methods” for details). The crystal structure shows that DG014 binds as expected with the phosphinic group at the active site, but with only the first five residues sufficiently structured to allow confident modeling (Fig. 2a, b). Ordered electron density of sufficient volume to contain ~2 amino-acid residues was observed 22 Å away from the active site, but we could not confidently ascertain whether this corresponded to buffer components or disconnected peptide C-terminal density, both of which have been observed at similar locations in domain IV in other crystal structures^19,21,22,30^.

Relative to previously determined structures of the open conformation for ERAP1 bound to bestatin^12^, conformational changes induced by peptide binding are apparent. A region between helices 4 and 5 (residues 426–433) that was not visualized in previous open conformation structures^11,12^ is now ordered and forms a short helix 4a (Fig. 2c). Helix 4a is in an intermediate state in the open DG014 structure relative to closed-form structures, with a shift required to adopt the configuration present in the closed conformer (Fig. 2d). In the closed state, phenylalanine 433 makes substantial contacts with bound ligand, as observed previously in the closed-state structure of ERAP1 bound to bestatin^11^, and conversion between the DG014-bound (open) and DG013-bound (closed) structures requires translation and rotation of helix 4a to bring phenylalanine 433 in contact with substrate P1 side chain (Fig. 2d).

Reorientation of Phe433 and motion of the helix 4a is coupled to rotation and tilt of helix 5, which repositions the side chain of Tyr438 (Fig. 2e). Based on mechanistic studies of M1 family aminopeptidases^31^ and mutagenesis studies of ERAP1^12^, Tyr438 is expected to play an important role in the catalytic mechanism by stabilizing the oxyanion that forms in the transition state with water attack on the scissile bonds of a peptide substrate. It was noted previously that Tyr438 near the end of helix 5 is oriented away from the substrate in the open conformation, but moves towards the active site in the closed conformation^11,12^. In the ERAP1–DG014 (open) complex, (Fig. 2e, cyan) as in previous open ERAP1 crystal structures, Tyr438 is oriented away from the active site and moves to interact with the peptide bond transition-state mimic phosphinic group in the ERAP1-DG013 (closed) complex (Fig. 2e, magenta), in a position analogous to the previously determined bestatin-bound ERAP1 closed structure^11^. Although this motion of tyrosine 438 into the active site previously has been associated with domain closure^12,13^, the coupling mechanism has been obscure. We suggest that the region between helices 4 and 5 (residues 426–433) acts as a sensor for substrate binding, ordering to form helix 4a in the presence of sufficiently long peptide-like substrates, and providing a site by which domain closure can be coupled to active-site reorganizations required for catalysis.

We note that helix 5 is linked to helix 4 through a disulfide bond between residues Cys443 and Cys404 (Fig. 2f), and that helix 4 forms the edge of domain II, where it lies along helices 10 and 12 at the edge of domain IV (Fig. 2g). The disulfide bond between helix 4 and helix 5 is unique to ERAP1 and ERAP2 among the M1 family of zinc aminopeptidases and is highly evolutionarily conserved among ERAP1 orthologs (Supplementary Fig. S6). For IRAP and CD13, two other M1 family members that have been crystallized in both open and closed conformations, helix 4 moves between open and closed states in conjunction with domain IV as is found in ERAP1. However, in these proteins, which lack a disulfide corresponding to Cys443-Cys404 in ERAP1, helix 4 motion does not couple with motion of the N-terminus of helix 5 (Supplementary Fig. S7). To test the role of this disulfide in ERAP1 in coupling domain closure with enzymatic activation, we introduced cysteine-to-serine mutations C404S and C443S to disrupt disulfide bond formation and measured the effect on enzymatic activity. The C404S and C443S mutations resulted in a significant decrease in peptide hydrolysis activity (Fig. 2h). To explore if this reduction was due to reduced coupling of domain closure and enzymatic activation, we examined the cooperativity of Leu-pNA hydrolysis. Fitting L-pNA hydrolysis data to an allosteric Michaelis–Mention model^12^ (see “Methods” for details), showed that *K*_half_ was not affected, indicating that the mutations did not affect substrate binding, but *k*_cat_ was significantly reduced, indicating an effect on the enzymatic processes subsequent to substrate binding (Fig. 2i). The Hill coefficient was reduced by disulfide bond disruption, as expected if it helped to couple allosteric site binding to active-site rearrangement (Fig. 2i). To critically test the role of the C404–C443 disulfide in coupling domain closure to enzymatic activation, we tested the effect of C404S and C443S mutation on allosteric activation induced by compound **3** (Supplementary Fig. S1), a previously characterized non-peptidomimetic small-molecule allosteric modulator^20^. Compound **3** activates ERAP1 for Leu-AMC hydrolysis; the activation has been hypothesized to act by stabilizing the closed conformer^20^, and this is confirmed below. The C404S and C443S mutations abrogated the allosteric activation effect of compound **3** (Fig. 2j). These results support a role for the C404–C443 disulfide in coupling binding site occupancy to enzymatic activation via domain closure.

### Peptide C-terminal binding sites localize within domain IV at sites accessible in the open conformation with additional contacts formed upon domain closure

**Fig. 3:**
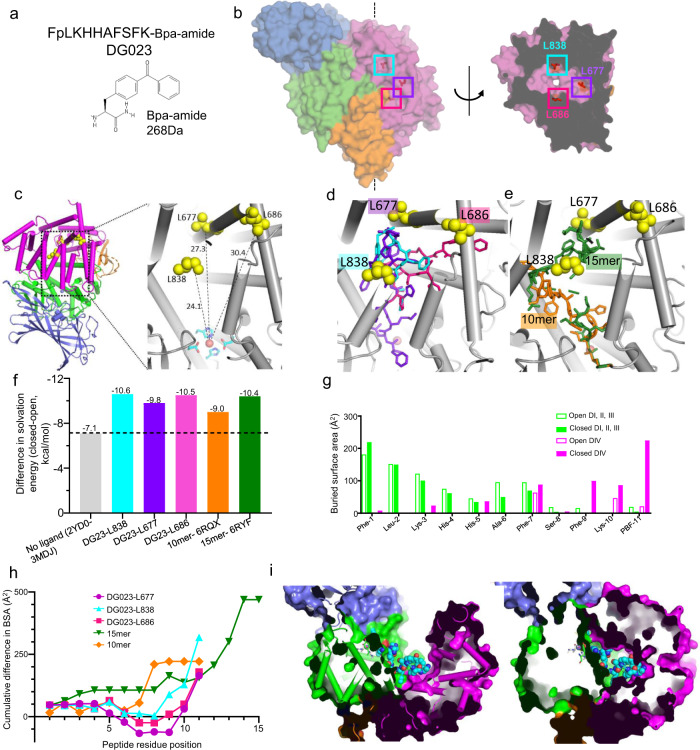
Binding of substrates and inhibitors increase interactions between domains II and IV. **a** Crosslinker peptide sequence is based on the sequence of characterized ERAP1 substrate^8^. First two amino acids are phenylalanine and leucine analogs connected by a phosphinic group “p”. Peptide C-terminus bears a photocrosslinker *p*-benzoylphenylalanine (BPA). After cross-linking, trypsin cleaves probe peptide leaving BPA-amide as a 268 Da adduct at the crosslink site. **b** Cross-linked ERAP1 residues reside in domain IV (magenta). Orthogonal views shown of closed ERAP1 structure (PDB ID 2YD0). Cross-linked amino acids shown as sticks with colored boxes. On right, a cutaway view reveals cross-linked sites in domain IV. **c** Close views of BPA-cross-linked ERAP1 Leu838, 677 and 686 localized in domain IV and their distances from the active site Zn is 24.1, 27.3 and 30.4 Å, respectively. **d** Close views of the paths of modeled DG023 peptide to each cross-linked site. **e** Paths of 10-mer (orange) and 15-mer (green) phosphinic peptides bound to ERAP1^21^ include L838 and L677 cross-linking sites. **f** Solvation energy of the closed conformer increases on binding long peptide substrates as compared to open conformer. 2YD0 (closed) and 3MDJ (open) were used as controls because of the absence of long peptides in the structures. DG023 peptide models and crystal structures of ERAP1 bound with 10-mer and 15-mer were used to calculate the solvation energy of open and closed conformers using PISA. **g** Buried surface area (BSA) of peptide residues in domain I, II, III (green), and domain IV (magenta) in open (open bars) and closed conformer (closed bars) along the length of the peptide. N-terminal peptide residues are more buried in domains I, II, III in both open and closed conformers while C-terminal peptide residues make more contacts in domain IV on its closure. BSA of the DG023-L838 peptide model is plotted here. **h** Cumulative difference in BSA along the length of the peptide between closed and open conformer increases for the C-terminal residues for peptide substrates. **i** Cross-sectional view of ERAP1 with DG023-L838 peptide model bound to open (left) and closed (right) conformation shows an increased number of contacts for the peptide C-terminal with domain IV in closed conformer (right). Source data are provided as a Source Data file.

ERAP1 exhibits side chain specificity at the C-terminal as well as N-terminal end of peptide substrates^17,19,21^, suggesting contact(s) between enzyme and substrate beyond the five residues of DG014 for which ordered peptide density was observed. A binding site for C-terminal aliphatic amino-acid residues has been identified within the interior of ERAP1’s substrate-binding cavity in a crystal structure of a substrate peptide bound to ERAP1’s isolated C-terminal domain^22^, and in a recent structure of intact ERAP1 in closed conformation bound to a long phosphinic peptide inhibitor^21^. Although aliphatic residues are preferred at the C-terminus of peptide substrates, peptides with other C-terminal amino acids also can be processed efficiently^8,17,32^. We used a photo-cross-linking approach to identify additional C-terminal binding site(s) for bound peptide substrates. We designed an 11-mer cross-linking probe, designated DG023, with peptide sequence based on a known ERAP1 substrate^8^, the nonhydrolyzable phosphinic group replacing the first peptide bond, and the unnatural amino-acid *p*-benzoyl-l-phenylalanine (BPA)-amide at the C-terminus^33^ (Fig. 3a). UV irradiation (350 nm) of BPA generates a carbene that can form a covalent bond with nearby molecules^33^. We performed cross-linking reactions in this manner using DG023 and purified ERAP1 and identified three cross-linking sites by LC-MS/MS (Fig. 3b, Supplementary Fig. S8a, b, see “Methods” for details). Two of the sites, Leu677 and Leu838, lie in domain IV close to the C-terminal aliphatic amino-acid residue binding site identified X-ray crystallography^11,22,34^ (Fig. 3b, c). The third site, Leu686, borders domains II, III, and IV at the in silico docking site identified for compound **3** (Fig. 3b, c); the homologous site is accessed by long peptides bound to mammalian aminopeptidase N (CD13), a structural homolog of ERAP1 involved in neuropeptide processing^35^. We were able to build conceptual models with good geometry for DG023 peptides extending from the active site Zn to each of the cross-linking sites, using the DG013 and DG014 structures as guides (Fig. 3d). Recent crystal structures of 10-mer and 15-mer phosphinic peptides reveal binding sites in the same general region in the interior of ERAP1’s substrate-binding cavity (Fig. 3e)^21^.

We performed buried surface area and solvation energy calculations for each of the structural models, shown in Fig. 3d, e, to help understand the impact of binding long peptide substrates on domain closure. Comparisons of open and closed conformers for ERAP1 bound to short inhibitors or peptides show that the closed structure has many additional interdomain contacts, mostly between domains II and IV, involving 77 residues (Supplementary Tables 2 and 3), and resulting in ~2800 Å^2^ of protein surface area accessible to solvent in the open conformation but excluded from solvent in the closed conformation. Such “buried” surface area correlates with greater energetic stability by decreasing the energetic requirement of solvation^36^. For ERAP1, domain closure results in a reduction of solvation energy between open and closed conformers estimated to be ~7 kcal/mole, calculated for ERAP1 crystal structures with the short peptide-like inhibitor bestatin (Fig. 3f, gray bar). Binding of long peptides creates additional sites for interdomain interaction, corresponding to an additional 355–1019 Å^2^ of protein surface area buried by domain closure (Supplementary Fig. S9a), and increased stabilization of the closed conformer by 1.9–3.5 kcal/mole (Fig. 3f, colored bars). ERAP1 makes contact with the bound peptides all along their lengths, but the contacts do not substantially involve domain IV, or contribute to the overall stabilization of the closed conformer, until the peptide extends beyond 6-9 residues, as shown in detail for the ERAP1–DG023-L838 model (Fig. 3g, see Supplementary Fig. S9b–e for the other complexes). A similar length dependence was observed for all the peptide models (Fig. 3h). A cross-sectional view of the ERAP1–DG023-L838 model illustrates how long peptide binding creates many sites for stabilization of the closed conformation (Fig. 3i, see Supplementary Tables 2 and 3 for the other complexes).

### An allosteric small-molecule activator induces ERAP1 domain closure

**Fig. 4:**
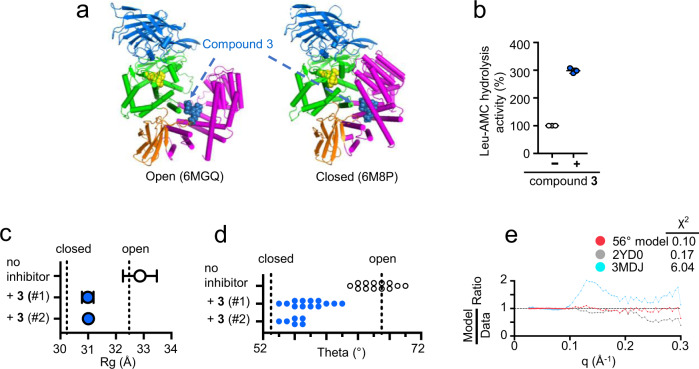
Allosteric small-molecule activator compound **3** induces ERAP1 closure. **a** Preferred binding pose for compound **3** at an interdomain interface distal to the active site^20^. **b** Compound **3** activates hydrolysis of dipeptide substrate leucine-amidomethylcoumarin (Leu-AMC). **c**–**e** ERAP1 adopts closed conformation in the presence of compound **3**, as measured by (**c**) *R*_g_ analysis, and (**d**) fitting to MD models. **e** Residual plot of best structural model fit to SAXS data in the presence of compound **3**, with crystal structure model fits shown for reference. Each data point in (**c**) represents a single independent experiment with *n* = 1 and the error of linear fit for each independent experiment is shown as error bars. Source data are provided as a Source Data file.

To investigate the relationship between conformational states and allosteric modulation of ERAP1 enzymatic activity, we characterized ERAP1 bound to the non-peptidomimetic small-molecule allosteric modulator compound **3** mentioned above. Compound **3** docks in a pocket at the junctions of domains II, III, and IV formed by the closed conformation of ERAP1 (Fig. 4a), and acts as an allosteric activator of ERAP1 by increasing hydrolysis activity towards the dipeptide substrate Leu-AMC^20^ (Fig. 4b). Compound **3** induced ERAP1 to adopt a closed conformation in solution, as shown by SAXS/WAXS *R*_g_ analysis (Fig. 4c) and by model fitting full scattering curves to those calculated for MD models as just described (Fig. 4d, e). *R*_g_ values and theta opening values were similar to those observed for ERAP1 bound to DG013 and the other small-molecule inhibitors. These results indicate that an allosteric activator can induce a similar closing motion as substrate binding and associate the closed conformation with the ‘active conformer’ hypothesized to explain the allosteric activation in other contexts.

### Position 528 Lys/Arg polymorphism alters domain closure energetics

Numerous genome-wide association studies (GWAS) have linked a common ERAP1 polymorphism at position 528 with susceptibility to inflammatory conditions of autoimmune etiology such as psoriasis, Behçet’s disease, and most strongly, with ankylosing spondylitis^5,37,38^. The disease-correlated variant codes for lysine at this position, compared to arginine in the protective *ERAP1* allele. This seemingly conservative substitution nevertheless has an effect on immune function, presumably by altering the presentation of peptides that stimulate an autoinflammatory response^7^. Proposed mechanisms for how the polymorphism influences ERAP1 activity include alterations in protein expression^39^ or altered protein conformational flexibility^18^. As reported previously^8,18^, we find that Arg528 distinctly alters hydrolysis activity of the dipeptide substrate Leu-pNA compared to Lys528, slightly increasing the enzyme catalytic rate *k*_cat_ but substantially decreasing the apparent substrate affinity as measured by increasing *K*_half_ (Supplementary Fig. S12).

**Fig. 5:**
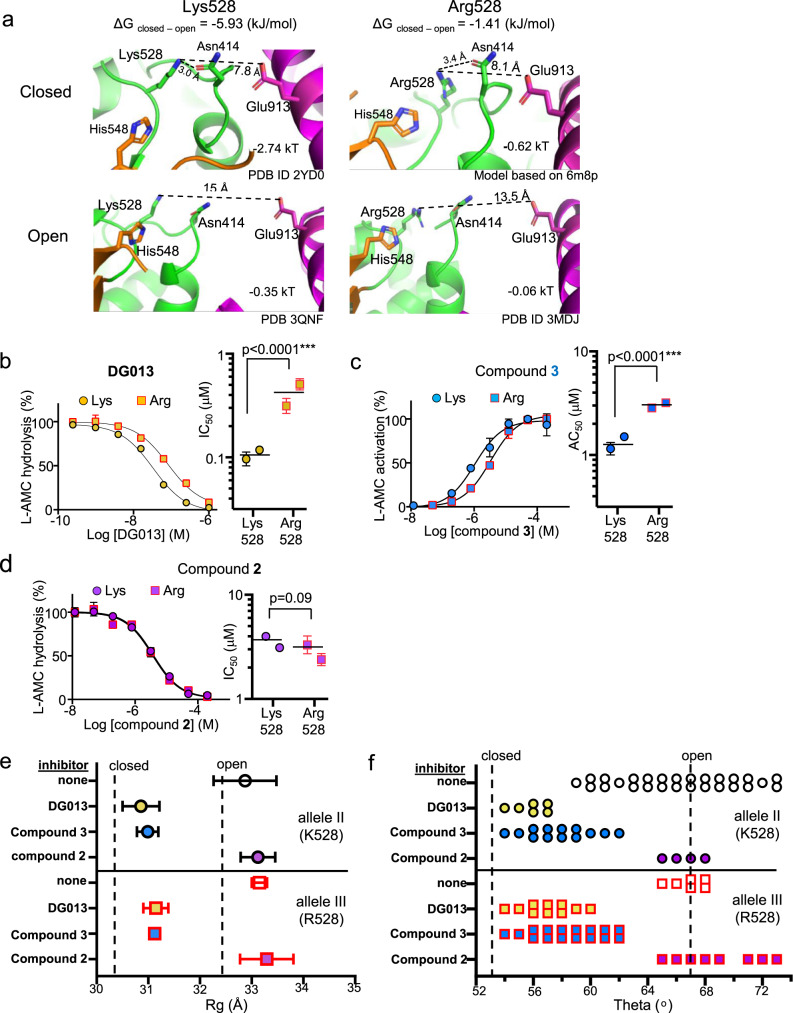
K/R polymorphism at position 528 modulates a conformationally dependent interdomain electrostatic interaction and regulates binding of small-molecule ERAP1 modulators that induce closing. **a** Lys or Arg at position 528 makes a long-distance electrostatic interaction with Glu913, in addition to interactions with Asn 414 and His 448. Open and closed conformations for proteins with Lys528 or Arg528 are shown. The closed Arg528 conformation is modeled based on 6M8P, all others are from crystal structures as indicated. Closest interatomic distances between Lys/Arg528 and Glu913 are 7.8 Å (closed) and 13.5 Å (open), respectively. Lys528 stabilizes the closed ERAP1 conformation more than does Arg528. Pairwise electrostatic potentials between residues 528 and 913 shown in kT units for each structure, calculated using DelPhiForce. **b** DG013 exhibits greater potency for Lys528 ERAP1 than Arg528 ERAP1. Leucine-AMC hydrolysis rate was measured and normalized to control condition without DG013. Data were fit to a sigmoidal curve with constrained top and bottom = 100 and 0% activity respectively. Significance calculated using two-tailed ANOVA (*P < 0.0001). **c** Compound **3** exhibits greater potency for Lys528 ERAP1 than Arg528 ERAP1. L-AMC hydrolysis rate was measured and normalized to control condition without compound **3**. Data were fit to a sigmoidal curve with constrained bottom = 100% activity. Significance calculated using two-tailed ANOVA (***P < 0.0001). **d** Compound **2** does not show ERAP1 variant-specific potency. Leucine-AMC hydrolysis rate was measured and normalized to control condition without compound **2**. Data were fit as in (**b**). For binding studies with all the three small molecules (**b**–**d**), two independent experiments were performed with triplicate (*n* = 3) samples in each experiment and variation between the replicates are represented by error bars. For both Lys528 (allele II) and Arg528 (allele III) variants, ERAP1 conformation remains open in the absence of ligand and in presence of compound **2**, as shown by *R*_g_ (**e**) or SAXS/WAXS model fitting (**f**). Both Arg/Lys528 variants of ERAP1 remain closed in the presence of compound **3** and DG013, as shown in the same analysis in (**e**) and (**f**). A single independent experiment (*n* = 3) was performed for *R*_g_ analysis on Lys528 and Arg528 variants of ERAP1 and error of linear fit for each independent experiment is shown as error bars (**e**). Source data are provided as a Source Data file.

Examination of the ERAP1 structure and electrostatic calculations shows that a positively charged Arg or Lys at position 528 forms a long-range electrostatic interaction with Glu913 in domain IV (Fig. 5a, long dashes). For both Arg528 and Lys528, the interaction would contribute preferentially to the closed conformation, as the pair of amino acids moves far apart in the open conformation (Fig. 5a, top panel). Calculation of the electrostatic potential between positions 528 and 913 using DelPhi shows that Lys528 has a stronger energetic contribution to the closed conformation than does Arg528, suggesting that it stabilizes the closed relative to the open conformation of ERAP1. The energetic difference as estimated by DelPhiForce is ~4.5 kJ/mol. Besides the Glu913 electrostatic interaction, Arg528 and Lys528 also interact with Asn414 and His548, in domains II and III, respectively^40^. Similarly, to the Glu913 interaction, the Arg/Lys528 interactions with Asn414 and His548 contribute preferentially to the closed conformation, with more favorable geometry for Lys528 as compared to Arg528 (Fig. 5a). Thus, through the long-range electrostatic interaction with Glu913 and interactions with Asn414 and His548, Lys528 would appear to stabilize the closed conformation relative to Arg528.

To investigate the effect of the 528 Arg/Lys polymorphism on the ability of ERAP1 to adopt the closed conformation, we measured the concentration dependence of ERAP1 modulation by two compounds that induce ERAP1 closure, the pseudopeptide active-site inhibitor DG013 (Fig. 5d) and the allosteric activator compound **3** (Fig. 5b, c), using a Leu-AMC hydrolysis assay. We used two allelic variants that differ only at position 528 (Supplementary Fig. S1). We reasoned that if the predicted electrostatic stabilization of the closed conformer of Lys528 ERAP1 over Arg528 ERAP1 were correct, compounds binding to the closed conformer should show preferential binding to Lys528 ERAP1. That is what was observed. Both compounds exhibited a higher potency for Lys528 ERAP1 (circles) than for Arg528 ERAP1 (squares); with lower IC_50_ for the inhibitor DG013 (Fig. 5b), and lower AC_50_ for the activator compound **3** (Fig. 5c). A similar polymorphism-dependent difference in inhibitory concentration has been reported previously for bestatin^41^, which also stabilizes the closed conformation of ERAP1 (Supplementary Fig. S2a). This behavior can be contrasted with that observed for compound **2** (Supplementary Fig. S1), a small-molecule inhibitor of ERAP1 that preferentially docks to the open conformer^20^, and does not induce ERAP1 domain closure, even when present at saturating concentrations (Fig. 5d, f, purple symbols). Unlike compound **3** and DG013, compound **2** exhibits equivalent potency for the two ERAP1 lysine/arginine 528 variants (Fig. 5d).

To investigate the effect of the position 528 Arg/Lys polymorphism on the ERAP1 open and closed conformations, we measured SAXS/WAXS scattering profiles. In the absence of ligand, the two ERAP1 variants are not distinguishable by Guinier Rg analysis (Fig. 5e, open circle and open square for Lys528 and Arg528, respectively) or by fitting scattering profiles to MD simulation-derived structures with different theta values (Fig. 5f, same symbols as 5e). In the presence of saturating concentrations of the inhibitor DG013 or the allosteric modulator compound **3**, both ERAP1 variants adopted the closed conformation (Fig. 5e, f). *R*_g_ and theta values were identical for the two variants. Thus, the 528 Arg/Lys polymorphism does not appreciably alter the conformation of ERAP1 in either the open or closed forms, despite its apparent effect in regulating the conversion between them.

Position 528 is neither located near the ERAP1 active site nor the docking sites for compounds **2** or **3**. Thus the greater effects of lysine/arginine 528 substitution on the binding of compounds that stabilize the closed over the open conformer are consistent with a model in which ERAP1 domain closure is altered by polymorphic variation at position 528, with arginine at position 528 impairing ERAP’s ability to dynamically adapt the closed form. Interestingly, similar effects on enzymatic parameters and DG013 binding were observed previously when ERAP1 conformational equilibrium was disrupted by mutations that destabilize ERAP1 interdomain salt bridges present in the closed conformation^28^.

## Discussion

These results, taken together, provide a comprehensive view of large-scale domain closure motions in ERAP1 and their role in catalytic function. ERAP1 in solution adopts a largely open conformation in the resting state, as shown by small-angle X-ray scattering (Fig. 1). Under these conditions, the opening angle theta^28^, which describes the orientation of domain IV relative to domains I, II, and III, is similar to that of the open conformer observed in crystal structures. In this conformation, the substrate-binding site is accessible to solution, but it is not configured for efficient processing, with interhelical loops disordered and a key active site tyrosine not optimally oriented for catalysis^11,12^. Binding of a variety of ligands including allosteric activators and peptide-like inhibitors promotes domain closure, in each case stabilizing a form with theta angle similar to that observed crystallographically for the closed conformer (Figs. 1 and 4). A complex of ERAP1 bound to the long peptidomimetic inhibitor DG014 trapped crystallographically in the open conformation exhibited some of the conformational attributes previously observed in closed-state structures, in particular ordering of helix 4a, suggesting that these changes were coupled to substrate binding (Fig. 2). Other changes associated with the closed conformation appeared to require domain closure, including the key rotation of helix 5 that brings Tyr438 into position for catalysis. Peptide models extending from the catalytic zinc to C-terminal binding sites identified by chemical cross-linking, as well as two recent crystal structures of ERAP1 bound to 10-mer and 15-mer peptides^21^, showed that bound peptides provide many sites for interdomain interaction, with residues beyond positions 7–9 contributing preferentially to stabilization of the closed conformer (Fig. 3). This observation suggests that domain closure will be more efficient for longer peptides because of the larger number of sites available for interdomain interaction and that this effect can provide the basis for ERAP1’s striking length-dependent “molecular ruler” cleavage activity^16,17^.

Early ideas about structural determinants for ERAP1 conformational change and allosteric regulation focused on a single regulatory site postulated to be accessible to peptides of eight residues or longer^12^. Occupancy of this site, for example by the C-terminus of sufficiently long peptides, or by allosteric activators, was hypothesized to trigger a conformational change to the closed conformation^12^. Subsequent structural work identified the binding sites for a peptide subtrate^22^ and a small-molecule allosteric modulator (compound **1**^19,20^), and the location of C-terminal residues for 10-mer and 15-mer phosphinic peptide inhibitors^21^ (Supplementary Fig. S10). Subsequent docking studies revealed a distinct location for a different allosteric activator (compound **3**^20^), identified also as the binding site for a buffer component from the crystallographic mother liquor^30^, and along the path for a long peptide exiting the substrate-binding cavity of the homologous protein human aminopeptidase N^35^ (Supplementary Fig. S10). The cross-linking studies reported here reveal that the C-terminus of bound DG023 peptide can access both of these regions in solution (Supplementary Fig. S10). ERAP1 has wide specificity for peptides of different sequences, with different peptide sequences exhibiting different internal and to a lesser extent C-terminal sequence preferences^17,42^, and this would appear to require degenerate binding modes. The surface area and energetic calculations reported here indicate that bound peptides in a variety of conformations can stabilize the active, closed conformation of ERAP1. Based on these results, we suggest that the large interdomain interaction surface comprising >70 residues provides many opportunities for stabilization of the closed form by allosteric activators and by long peptides that can bridge between domains II and IV, regardless of the exact path of the bound peptide (Supplementary Tables 2 and 3).

A recent analysis of ERAP1 molecular dynamics trajectories calculated from high-resolution closed-conformer crystal structures revealed a path of correlated dynamic motions linking Tyr438 in the active site with residues near the C-terminus of the bound 15-mer peptide^21^. The network path was shorter in the presence of an N-terminally bound tetrapeptide, suggesting that substrate binding results in stronger dynamic coupling between active and regulatory sites^21^. Our results help to confirm and extend this model. The C404–C433 disulfide, required for efficient allosteric activation, lies along the proposed path (Supplementary Fig. S11), and provides a mechanism for at least some of the proposed conformational coupling. Perhaps more importantly, we note that in the open conformation the path of correlated motions is broken because of the movement of domain IV helices 10 and 12 relative to domain II helices 4 and 5 (Supplementary Fig. S11). Thus, domain closure would appear to be required for efficient conformational coupling of regulatory and active sites via the proposed network path.

Electrostatic stabilization of the closed conformer of ERAP1 provides a mechanism for the observed effects of the key Lys/Arg528 polymorphism. Of the nine common ERAP1 single amino-acid polymorphisms^43^, K528R is the most strongly associated with autoimmune disease^4,5,43^, with substantial effects on enzymatic activity toward both single-residue and peptide substrates^8^. The K528R amino-acid substitution is conservative and located away from the catalytic center and substrate binding cavity, and thus the mechanism by which Lys/Arg528 impacts ERAP1 activity has been an outstanding question in the field. Some other common polymorphic sites are located within the interior compartment, in position to interact with a bound peptide^12^, or along the path of coupled motions^21^, but position 528 is on the surface of the protein at the junction of domains II, III, and IV). Crystal structures of ERAP1 show that lysine or arginine at this position can participate in a long-range electrostatic interaction with Glu913 in domain IV when the protein is in the closed but not the open conformation (Fig. 5a). Poisson–Boltzmann electrostatic calculations suggest that this interaction stabilizes the closed form. In addition, an intradomain interaction involving Lys/Arg528, Asn414, and His548 and specific to the closed conformation is more favorable in the Lys528 variant, as previously noted^40^, also tending to stabilize the closed conformer. The energetic effects of these changes are predicted to be relatively small, and we did not observe any substantial differences in the SAXS profiles of K528 and R528 that would have been indicative of substantial alterations in the equilibrium mixture of open and closed forms, or a change in the extent of interdomain motion in the open form. However, we did observe significant differences in the potency of two inhibitors that bind at two different sites and induce domain closure (Figs. 1 and 4). Both compounds were more potent for the K528 variant, consistent with the proposed electrostatic stabilization of the closed form for this variant and preferential binding of the inhibitors to binding to the closed form. Previous molecular dynamics simulations also suggested a role for the K/R528 polymorphism in regulating ERAP1 conformational dynamics and domain motions, with K528 shifting the conformational ensemble towards the closed conformation^18^. As ERAP1 inhibitors are increasingly being considered as potential therapeutics for cancer immunotherapy and autoimmunity, the effect of common ERAP1 polymorphisms on inhibitor potency must be considered. With regard to understanding the role ERAP1 polymorphism in autoimmune etiology, stabilization of the closed form (as observed for K528) would tend to promote increased activity under conditions where substrate binding and/or domain closure were limiting, but for some substrates turnover might be limited by product release or other aspects of the enzymatic cycle involving the open conformer. In these cases, the greater relative stability of the closed conformation might be detrimental to the overall activity. Thus, the effects of the ERAP1 polymorphism and domain closure on processing particular candidate autoimmune epitopes^6,7^ might be difficult to predict.

Several questions and uncertainties about ERAP1 enzymatic mechanism and regulation remain. Solution-scattering studies were highly informative in relating ERAP1 conformational states in solution to those observed in crystal structures, and in revealing that binding of substrates, inhibitors, and allosteric activators all induce domain closure from a resting open conformation. However, an important caveat of using SAXS to study conformational states in this system is the difficulty in distinguishing mixtures of states from single conformations. Although in principle information about the distribution of conformational states is available from SAXS studies, we observed similar residuals in fits of experimental scattering curves to single conformations and to mixtures of conformations. For each experimental condition, we identified an ensemble of individual conformations consistent with the experimental data, but whether the individual conformations represent actual species or weighted averages of open and closed forms is not clear. Moreover, we only considered conformations observed in crystal structures or accessible by standard or accelerated molecular dynamics simulations starting from the crystal structures^18,28^. Model-independent approaches to SAXS data analysis such as low-resolution envelope or bead-model generation^44^ potentially could identify novel conformations present in the solution ensemble. We expect that ERAP1 molecules transition throughout an energy landscape between open and closed low-energy states, as suggested by molecular dynamics simulations, and with the ensemble of molecules in solution populating these states and any intermediates therein in a dynamic equilibrium. Several lines of evidence support the dynamic nature of the conformational equilibrium, including the crystallographic trapping of ERAP1 in the open state with inhibitors like DG014 and bestatin despite predominance of the closed form in solution, and the allosteric promotion of enzymatic cycling by allosteric activators that stabilize the closed form. Another question relates to the relationship of domain closure to the catalytic cycle. Further studies will be required to elucidate the kinetics of domain closure, and whether these match the catalytic turnover rate, which is ~ 1–10 s^−1^ for optimal substrates under *V*_max_ conditions^8,20^. ERAP1 can process peptides as long as 25 residues^17,32^, albeit inefficiently, and these would appear to be too long to fit completely in the substrate-binding chamber, particularly if they need to access any of the currently identified C-terminal binding sites. Potentially, such peptides could be accommodated with their C-termini outside of the site, exiting the chamber in the vicinity of Leu686 where helix 4 contacts helix 12, at the domain II/IV junction, as does substance P bound to the ERAP1 structural homolog APN (Supplementary Fig. 10). However, this binding mode would preclude complete domain closure without substantial reorientation of helix 4 and/or helix 12. Whether partial closure as observed for APN would be sufficient to induce active-site rearrangements allowing catalysis remains to be determined. A final caveat to the model presented here relates to the steep increase in ERAP1 processing rate observed as the peptide length increases beyond 8 or 9 residues^12,17^. The stabilization of the closed conformation due to the accumulation of interdomain contacts mediated by bound peptide substrate also increases with peptide length beyond ~7 residues (Fig. 3h), but the increase is more gradual. This would suggest that additional slow steps associated with domain closure remain to be identified, such as the ordering of interhelical loops disordered in the open form^11,12^. These would help to stabilize to closed conformer upon domain closure and synergize with C-terminal binding to promote the network of correlated residue motions^21^ leading to the formation of the catalytically competent active-site configuration.

## Methods

### Mutagenesis and baculovirus production

Sequence variants of ERAP1 were generated (inserted in pFastBac plasmid) using Agilent QuikChange II XL site-directed mutagenesis. Primers used for site-directed mutagenesis of ERAP1 are listed in Supplementary Table 4. All ERAP1 variants were validated by sequencing the entire open reading from in the expression plasmid to confirm identity and lack of unexpected mutations. Positive clones were used to make bacmids and baculoviral stocks were prepared using SF9 insect cell line (CRL-1711, ATCC) following Bac-to-Bac baculoviral system protocol.

### Protein expression and purification

High Five cells (BTI-Tn-5B1-4, CRL-10859, ATCC) were infected with baculoviral stocks and cultured for three days at 27 °C. Cells were then pelleted, and the supernatant was concentrated to ~100 mL and buffer exchanged >100-fold into 50 mM Tris pH 8, 300 mM sodium chloride, 10 mM imidazole. Samples were bound to Ni–NTA–agarose resin, washed with 10 mM imidazole buffer, then washed with 20 mM imidazole buffer, then eluted with 100 mM imidazole buffer. Samples were then purified by anion-exchange chromatography and size-exclusion chromatography and stored at −80 °C until use. The purity and integrity of all purified proteins were analyzed by SDS-PAGE with Coomassie Blue R250 staining (Supplementary Fig. S1b). For purification of C404S and C443S ERAP1 mutants, 1 mM DTT was kept through all the steps to prevent oxidation of the free cysteine. For activity assays of these mutants and wild-type ERAP1, a comparable amount of DTT was added to each sample.

### Chemical synthesis

#### Synthesis of phosphinic peptide DG013

The synthesis of phosphinic pseudopeptide DG013 has been described^29^.

#### Synthesis of phosphinic peptide DG014

Phosphinic pseudopeptide DG014 was synthesized by applying standard solid-phase peptide synthesis, on trityl alcohol lanterns (15 µmol/pin) using a Fmoc chemical protocol. A solution of acetyl chloride in dry dichloromethane (1:10 v/v) at room temperature was used to afford the trityl chloride lanterns. Attachment of the first amino-acid Fmoc-Tyr (tBu)-OH (30µmol/pin) was performed by using N,N-diisopropylethylamine (18 µL/pin) in dry dichloromethane (0.4 mL/pin) at room temperature for 12 h^45^. The loading amount of Fmoc-Tyr (tBu)OH was evaluated to be 12 μmol/pin, after cleavage from the polymer-support with 0.5% trifluoroacetic acid (TFA)/dichloromethane (room temperature, 1 h). Fmoc deprotection was performed with a solution of 20% piperidine in N,N-dimethylformamide over 1 h for each cycle of the synthesis. Fmoc protected amino acids (45 µmol/pin), 1-hydroxybenzotriazole (45 µmol/pin), and diisopropylcarbodiimide (45 µmol/pin) in dichloromethane/N,N-dimethylformamide (6/1) (0.4 ml/pin), were used for the coupling steps and each coupling reaction was allowed to proceed for 5 h. Coupling of the building block Boc- (R)hPhep[PO (OAd)CH_2_] (R,S)LeuOH (23µmol/pin)^29^ was performed using the coupling conditions described above (36 µmol/pin of each reagent). Deprotection and removal of the final pseudodecapeptide from the solid support was accomplished by using a solution of TFA/dichloromethane/triisopropylsilane/H_2_O 39/58/2/1 for 2 h at room temperature. After concentration in vacuo, the crude product was precipitated in cold diethyl ether. DG014 was obtained after purification by analytical RP-HPLC and characterized by mass spectroscopy [ESMS *m/z* (*z* = 1): calcd for [C_62_H_90_Ν_9_Ο_18_P + H] ^+^1281.4; found: 1281.5].

#### Synthesis of phosphinic peptide DG023

The phosphinic pseudoundecapeptide DG023 was prepared by conventional solid-phase peptide synthesis, on Rink amide lanterns (8 μmol/pin), using the Fmoc strategy. Fmoc deprotection and amino-acid coupling steps were performed as described above for DG014. After the introduction of Fmoc-Bpa-OH all synthetic steps were performed in light-protected conditions. For the introduction of phosphinic pseudodipeptidic sequence, the building block Boc- (R)Phe[PO (OAd)CH_2_] (S)LeuOH was synthesized in three steps starting from the R-stereoisomer of the Boc-protected aminophosphinic analog of phenylalanine^46,47^. The phosphinic pseudodipeptide Boc (R)Phe)[PO (OH)CH_2_] (S)LeuOEt was prepared as previously described^48^, and obtained in a stereochemically pure form after two recrystallizations with AcOEt. Subsequent adamantylation of the phosphinic group and saponification of the C-terminal ethyl ester group afforded the final building block Boc (R)Phe[PO (OAd)-CH_2_] (S)LeuOH^48^, which was incorporated in the last step of the solid-phase synthesis. For the coupling of the aforementioned building block, 16 µmol/pin were used by using standard coupling conditions. The final pseudoundecapeptide was cleaved and deprotected from the solid support in presence of a solution of trifluoroacetic acid (TFA)/H_2_O/triisopropylsilane 95/2.5/2.5 over 2 h at room temperature. The solution of deprotected peptide was concentrated in vacuo and the residue was treated with cold dry diethyl ether. DG023 was obtained after purification by analytical RP-HPLC and characterized by mass spectroscopy [ESMS *m/z* (*z* = 1): calculated for [C_79_H_104_Ν_17_Ο_14_P + H] ^+^1546.8; found: 1546.9].

### Small-angle X-ray scattering

Homogeneity of all ERAP1 samples used for SAXS studies was assessed the day before analysis by size-exclusion chromatography and SDS-PAGE. Samples were then stored at 4 °C until data collection. Samples used for SAXS studies were stored after data collection, and purity and integrity were confirmed a day after the data collection by SDS-PAGE. Just prior to analysis, purified protein samples were mixed with inhibitor, if indicated, and concentrated in rinsed Centricon 10 kDa MWCO 0.5 mL centrifugal concentrators equilibrated in 50 mM HEPES pH 7.5, 200 mM NaCl, 0.02% (w/v) NaN_3_. Concentrator retentate and flow through were stored and used as sample and buffer, respectively, during SAXS data collection. SAXS data were collected for each sample at three concentrations (generally 4 mg/mL, 2 mg/mL, and 1 mg/mL). Small-molecule additives were included at the following concentrations: 2 mM bestatin, 30 μM leucinethiol with 30 μM dithiothreitol, 120 μM SIINFEKL peptide, 100 μM DG013, 200 μM compound **3**, 200 μM compound **2**. Scattering curves were buffer subtracted using matched buffer-scattering curves. The concentration series was then compared, scaled, and merged manually using PRIMUS (in ATSAS v2.5.2) and SCÅTTER (version 3.0 g). Merged SAXS curve *R*_g_ was calculated using AutoRG. Minimal ensemble search was performed using the FOXS webserver^49^ with data fit to single-structural models of ERAP1.

### ERAP1 structural modeling

Structural models were generated by two methods. For models derived from crystal structures (PDB ID 2YD0, and 3MDJ chains A/B/C), missing portions of polypeptide and complete high mannose N-glycans were added by rounds of simulation using the AllosMod webserver^50^. Alternately, structural models were sampled from a prior molecular dynamics simulation of ERAP1^28^.

### Crystallization

Purified protein samples were prepared in 10 mM Tris or 10 mM HEPES buffer. Crystal trials were set up and hits were optimized. For DG013-bound ERAP1 cocrystal, final crystallization conditions were: 1.3 M ammonium sulfate, 100 mM MES pH5.5, 15 mg/mL ERAP1 preincubated with saturating DG013, grown at 25 °C, dehydrated by changing well solution to 2 M ammonium sulfate one day prior to looping, cryoprotected with lithium sulfate. For DG014-bound ERAP1 cocrystal, final crystallization conditions were: 15% PEG8000, 100 mM Tris pH 8.60, 3% d-sorbitol, 7.5 mg/mL ERAP1 preincubated with saturating DG014, grown at 4 °C, cryoprotected with ethylene glycol crystals were mounted on loops and frozen by plunging in liquid nitrogen in preparation for data collection.

### X-ray diffraction data collection and structure determination

Crystal diffraction data were collected in a 180° arc about a static crystal (PDB ID 6MGQ) or a 360° arc about a crystal transiting along a defined vector (PDB ID 6M8P). X-ray wavelengths used were 1.110 Å (PDB ID 6MGQ) and 0.979 Å (PDB ID 6M8P). Diffraction data were collected at 100 K. X-ray diffraction data were integrated using XDS version June 1, 2017^51^ with anisotropy assessed using STARANISO^52^. STARANISO assigns an anisotropic resolution cutoff, to account for different diffraction intensities in different directions in reciprocal space. For ERAP1–DG014 (PDB ID 6MGQ), anisotropy was minimal, and resolution limits in the best (2.9 Å along 0.40 a*+0.977 b* + 0.211 c*) and worst (3.5 Å along 0.427 a* + 0.885 b* + 0.183 c*) directions were similar. For ERAP1-DG013 (PDB ID 6M8P), anisotropy was substantial, with resolution limits in the best (3.3 Å along 0.262 a* + 0.965 b*) and worst (7.4 Å along 0.998 a* + 0.062 c*) directions substantially different. Table 1 reports overall and last-shell completeness for both conventional spherical cutoffs and also for the elliptical cutoffs used for refinement and map interpretation. Space groups were tentatively assigned using observed point group symmetry and patterns of systemic absences and then confirmed by molecular replacement trials in all possible space groups consistent with lattice symmetry using Phaser^53^, and by refinement tests in all potential subgroups and supergroups using Zanuda^54^. Resolution limits were assigned using a CC* cutoff of ~0.5^55^. Ligand occupancy was confirmed by unconstrained occupancy refinement for each molecule in the asymmetric unit, and by examination of omit maps^56^.

ERAP1–DG014 crystallographic space group and unit cell parameters were similar to a previously determined crystal structure for ERAP1 in the open conformation bound to bestatin (PDB ID 3MDJ)^12^, and the structure was determined by molecular replacement using those coordinates, searching separately for domains I + I + III and domain IV. The protein formed a trimer in the crystallographic asymmetric unit (Supplementary Fig. S3g, h), with domain organization slightly more open than for 3MDJ. The phosphinic peptide inhibitor DG014 chemical structure was generated using the PRODRG webserver^57^. Restraints were then generated using eLBOW^58^, and models were built through cycles of refinement using Phenix^53^ and manual rebuilding using Coot^59^. Omit maps were generated using Polder^56^. Electron density for the bound DG014 phosphinic peptide was very weak beyond the proline at residue 5. Additional density potentially corresponding to approximately two additional residues was observed 14 Å away in the vicinity of ERAP1 residues F405, D406, F674, Q675, N678, and M726. Full-length 10-residue models for DG014 with reasonable geometry would extend into this region, but the observed electron density did not support confident placement and refinement of a full-length peptide model. In the deposited PDB file, an unknown ligand record (UNL) was used to mark this location.

ERAP1-DG013 adopted a very large crystallographic unit cell, with Matthews coefficient consistent with up to 41 molecules of ERAP1 per asymmetric unit. Molecular replacement trials using ERAP open, closed, and isolated domain search models revealed strong solutions for 22 molecules of ERAP1 in the closed conformation, in an unusual arrangement of two stacked 11-membered rings (Supplementary Fig. S3b–d). *Z* scores from these solutions ranged from 27 (log-likelihood gain LLG = 632) for the 1st solution to 104 (LLG = 1876) for the second solution; the mean for all 22 solutions was 48 ± 19 (s.d.), with a final LLG of 92875. No other significant solutions were observed. The stacked rings aligned with their 11-fold axes of symmetry parallel to the unit cell a axis, and formed an open lattice (Supplementary Fig. S3). Only 16 of the 22 molecules in the asymmetric unit (8 from each stacked ring) participated in contacts in the b–c plane (Supplementary Fig. S3). The high solvent content (73%) and relatively low degree of intermolecular contacts resulting from this packing can in part help to explain the very weak diffraction observed for this crystal (overall I/σ(Ι) = 2.68, cf. I/σ(Ι) =20.11 for ERAP1–DG014). Despite the weak diffraction, clear omit map electron density was observed for each of 22 ERAP1 monomers (Supplementary Fig. S4). The 22-fold averaged map was used for the initial building of DG013 and rebuilding of the ERAP1 active site. Twenty-two copies of this complex were then placed appropriately in the asymmetric unit for cycles of refinement and rebuilding. Crystallographic refinements with 22-fold non-crystallographic restraints and conservative geometry resulted in a final refined model with working and free R-factors, RMS deviations from ideal geometry, and main-chain and side-chain torsions and clash scores (Table 1) well within the range of PDB entries with the comparable resolution, as assessed by MolProbity^60^. Final refined occupancies for each of the bound DG013 monomers were near unity (Supplementary Fig. S4). Refined atomic B-factors were relatively low (<B > 39.5 Å^2^), perhaps surprising for a crystal with such a low-resolution limit and a high degree of anisotropy, but were consistent with the low observed Wilson B-factor (29.6 Å^2^), and demonstrate the utility of TLS refinement^61^ in modeling anisotropic diffraction. The resolution limit of 3.3 Å was confirmed using a paired refinement test^55,62^; however, the effective resolution will be somewhat lower because of the anisotropic diffraction and loss of data beyond the elliptical resolution cutoff. Because of the relatively low resolution and weak diffraction observed for this crystal, we limited interpretation to localization of the bound DG013 ligand to the active site.

### Activity assays

ERAP1 activity was measured consistently in 96-well format, 100 μL reactions containing 20 mM Tris pH 7.5, 100 mM NaCl, 0.02% sodium azide, with 0.1% (w/v) bovine specific antigen (BSA) present to block ERAP1 binding to plastic (observed even for “non-binding” polypropylene plates). For peptide hydrolysis, ERAP1 (0.2 ng/μL) was mixed with inhibitor (25 μM) and then incubated with substrate peptide (10 μM) for 10 min. Reactions were stopped by addition of trifluoroacetic acid, 0.4% (v/v) final concentration. Plates were frozen and shipped to PureHoney Technologies for quantification of substrate and product by LC-MS and summing area-under-curve of respective peaks. For leucine-amidomethylcoumarin (Leu-AMC) hydrolysis, ERAP1 (2 ng/μL) was mixed with inhibitor if indicated and reactions were begun by addition of Leu-AMC substrate (100 μM). Product formation (7-amino-4-methylcoumarin) was quantified by measuring the change in fluorescence (380 nm excitation/460 nm emission) over 20 min at using BMG POLARstar OPTIMA. For measurements of peptide hydrolysis using coupled enzyme assay, 50 µL ERAP1 (400 ng/well) was mixed with the 25 µL substrate LF9 peptide (LVAFKARKF)^42^ at a series of concentrations along with 25 μL of coupling reagent to start the reaction as described before^20^. After starting the reaction, absorbance at 405 nm was measured once every 2.5 min for 20 min. Reaction slopes were quantified by calculating the absorbance change over the time course, which was linear under these conditions. Michaelis−Menten analysis was performed by fitting the slopes as a function of substrate concentration in standard Michaelis−Menten equation using GraphPad Prism.

### Photocrosslinking and mass spectrometry

We performed several optimization and control experiments to establish appropriate conditions for photo-cross-linking. ERAP1 DG023-inhibition studies were used to estimate the saturating concentrations for the cross-linking assay. IC_50_ for DG023 inhibition of Leu-AMC hydrolysis by ERAP1 was observed at 15 nM, similar to the other phosphinic peptides DG013 and DG014 (Supplementary Fig. S5). For the cross-linking experiment, 100 μM DG023 was used with 20 µM ERAP1; under these conditions, 99.4% inhibition of ERAP1 should be obtained. To test if BPA at the C-terminus of the peptide substrate interferes with the binding to ERAP1, we followed the hydrolysis of a non-phosphinic peptide substrate based on DG023 (LLKHHAFSFK-BPA). ERAP1 hydrolysis was similar to a known peptide substrate, LF9 (LVAFKARKF)^42^, (Supplementary Fig. S8d), indicating that BPA at the C-terminus did not interfere with ERAP1 binding. In preliminary experiments, different irradiation times (5, 10, 15, 20, and 30 min) were tested, with optimum cross-linking observed at 30 min. To evaluate any effects of UV exposure on the structure or activity of ERAP1, L-pNA hydrolysis activity was tested before and after 30 min of irradiation, and no difference was noted (Supplementary Fig. S8c). A cross-linking experiment performed with BPA alone instead of DG023 did not identify any adducts, indicating the absence of substantial non-specific modification by this cross-linking reagent. Purified ERAP1 was incubated in a V-bottom 96-well plate in the absence of any ligand, in the presence of BPA alone, or in the presence of DG023 cross-linking peptide inhibitor. Samples were irradiated for 30 min on ice using a long-wavelength UV lamp (Blak Ray B100AP/R, UVP), and then denatured, deglycosylated enzymatically, and separated by SDS-PAGE. Excised gel bands were trypsin-digested and analyzed by LC-MS/MS at the UMass Proteomic Core Facility. Crosslinks were detected by searching the SwissProt human proteome database (Uniprot_human proteome [https://www.uniprot.org/proteomes/UP000005640]) using Sequest (IseNode from Proteome Discoverer 2.1.1.21, fragment ion mass tolerance of 0.050 Da, parent ion tolerance of 10.0 PPM) for a + 268 Da modification on leucine, serine, tryptophan, or methionine. One peptide identified using these criteria with *m/z* consistent with a + 268 modification of an ERAP1 tryptic peptide was excluded from analysis as it appeared in the dataset generated in the absence of DG023 or BPA. Cross-linked peptides were identified with 75% peptide confidence or greater, and 2.74% prophet FDR (spectra). Data analysis and figure preparation were performed using Scaffold 4^63^.

### Electrostatic potential calculation

Structural models of ERAP1 domains II–IV (lacking domain I due to limits on the number of atoms allowed) were prepared for analysis using PDB2PQR, which calculated atomic charges using AMBER force field^64^. The output of PDB2PQR was then input to the DelPhiForce webserver^65^ to calculate the pairwise electrostatic potential. Settings used were: pairwise interactions mode, Gaussian dielectric distribution method, 3.0 grids/Å, 0.2 M salt concentration. To test the effect of substitution at position 528, computational mutagenesis was performed on existing structural models, using the respective residue rotamers found in the alternate ERAP1 crystal structure.

### Structural modeling and analysis

Previously determined crystal structures of ERAP1 bound to 10-mer and 15-mer phosphinic peptides were observed in the closed conformation (6RQX and 6RYF). DG023 peptides with C-terminal BPA crosslinker bound to L677, L686, and L838 were modeled into the closed ERAP1 conformation (2YD0) using Coot^59^. For solvent-accessible surface area calculations, open-conformer models with bound peptides were generated by superimposing peptide bound closed ERAP1 with open ERAP1 (3MDJ) using Pymol^66^ using the pair fit function, aligning the α carbons of ERAP1 domains. Long peptide substrates and Zn were considered to be part of domains I, II, III. Glycans and all other ligands and buffer components were removed from the crystal structures. The PISA server^67^ was used for surface area and solvation energy calculations, and for the identification of interdomain contacts. ERAP1 was divided into two different chains, where domains I, II, and II were considered to be one chain and domain IV the other chain. Residues identified in interdomain contacts for the closed (2DY0) but not open (3MDJ) conformation are shown in Supplementary Table II. Additional residues involved in interdomain contacts in the various long peptide structures and models are shown in Supplementary Table III.

### Statistics

For SAXS analysis, radii-of-gyration are presented as means of each curve fit with the respective fit error plotted. Fitting of SAXS data with structural models used chi-square to quantify goodness-of-fit as well as model/data ratios to qualitatively demonstrate residual deviation. Unpaired *t* tests were used to compare replicate experiments. For statistical analysis of EC_50_, sigmoidal curves were fit to eight concentrations of inhibitor, in triplicate. LogEC_50_, standard error of measurement, and the number of points (24) from two experiments were analyzed for statistical significance using two-tailed ANOVA.

### Figure design

Structural figures were prepared using Pymol^66^. Graphical data were prepared with GraphPad Prism 7.0c^68^.

### Reporting summary

Further information on research design is available in the Nature Research Reporting Summary linked to this article.

## Supplementary information

Supplementary Information

Reporting Summary

## Data Availability

The crystallographic diffraction data and atomic coordinates generated in this study have been deposited in the world-wide Protein Data Bank (wwPDB) under accession codes 6MGQ and 6M8P. The raw crystallographic datasets have been deposited in the SBGRID repository under accession codes 605 and 606. The other X-ray crystal structure coordinates used in this study are available from in wwPDB under accession codes 3MDJ, 3QNF, 2YD0, 6RQX, and 6RYF. The human proteome sequences used for mass spectrometry data analysis are available from the Uniprot database under accession code 5640.  Source data are provided with this paper.

## Source data

Source Data
