# Author Correction: Conformational dynamics linked to domain closure and substrate binding explain the ERAP1 allosteric regulation mechanism

**DOI:** 10.1038/s41467-023-37300-7

**Published:** 2023-03-20

**Authors:** Zachary Maben, Richa Arya, Dimitris Georgiadis, Efstratios Stratikos, Lawrence J. Stern

**Affiliations:** 1 Department of Pathology, University of Massachusetts Medical School, Worcester, MA USA; 2 Department of Chemistry, National and Kapodistrian University of Athens, Athens, Greece

**Keywords:** Enzyme mechanisms, SAXS, X-ray crystallography

Correction to: *Nature Communications* 10.1038/s41467-021-25564-w, published online 06 September 2021

The original version of this Article omitted the following from the Acknowledgements: “This research used resources of the Advanced Photon Source, a U.S. Department of Energy (DOE) Office of Science user facility operated for the DOE Office of Science by Argonne National Laboratory under Contract No. DE-AC02-06CH11357.” This has now been corrected in both the PDF and HTML versions of the Article.

