# Supplementary material for: Conformational dynamics linked to domain closure and substrate binding explain the ERAP1 allosteric regulation mechanism
Source: Nat Commun. 2021 Sep 6;12:5302. doi: 10.1038/s41467-021-25564-w (PMC8421391; doi:10.1038/s41467-021-25564-w)
Supplement: Supplementary file 1 — Supplementary Information [file 41467_2021_25564_MOESM1_ESM.pdf]

Supplementary information

ERAP1 domain closure is coupled to substrate binding, allosteric activation, and catalysis

*Zachary Maben [+1], Richa Arya [+1], Dimitris Georgiadis[2], Efstratios Stratikos[2], Lawrence J Stern [*1]*

[1] Department of Pathology, University of Massachusetts Medical School, Worcester MA, 01655, USA
[2] Department of Chemistry, National and Kapodistrian University of Athens, Panepistimiopolis, Zografou, Athens 15784, Greece
[+] These authors contributed equally to this work.
*Address correspondence and material requests to LJS (Lawrence.Stern@umassmed.edu)

Supplementary Table 1: List of interatomic contacts between ERAP1 and phosphinic inhibitors DG013 and DG014 in crystal structures.

Supplementary Table 2: List of residues involved in interdomain interactions induced by domain closure, identified in crystal structures of ERAP1 in open and closed conformations.

Supplementary Table 3: List of residues involved in additional interdomain contacts induced by domain closure observed in crystal structures and models of long peptide complexes.

Supplementary Figure 1: Sequences of ERAP1 constructs used in this work and Structural formulas of small molecules used in this work.

Supplementary Figure 2: SAXS data on substrates and inhibitors with ERAP1

Supplementary Figure 3: Overview of ERAP1-DG013 (open form) and ERAP1-DG014 (closed form) crystal structures.

Supplementary Figure 4: DG013 ligand omit-map electron density

Supplementary Figure 5: ERAP1 inhibition data for phosphinic peptide compounds DG013, DG014 and DG023.

Supplementary Figure 6: Sequence alignment of ERAP1 orthologs and paralogs.

Supplementary Figure 7: Cys403-443 disulfide couples active site rearrangements with interdomain interactions,

Supplementary Figure 8: MS/MS data for ERAP1 tryptic peptides modified by photoactivatable crosslinker.

Supplementary Figure 9: Solvent-accessible and buried surface area analysis of ERAP1 derived from PISA server.

Supplementary Figure 10: Peptide C-terminal and allosteric activator binding sites.

Supplementary Figure 11: ERAP1 domain motion and a network of correlated motions

Supplementary Figure 12: Disease-associated polymorphism at position 528 modulates ERAP1 enzymatic activity.

a

ERAP1 atom chain A	DG013 atom chain A	Interatomic distance (Å)
Glu 183 OE1	N09	2.5
Gly 317 N	O12	2.8
Glu 320 OE1	N09	3.0
Glu 354 OE2	O01	3.1
Glu 376 OE1	O02	2.9
Glu 376 OE2	N09	4.0
Lys 380 NZ	N36	4.0
Phe 433 CD1	C06	3.5
Phe 433 CE2	C05	3.5
Tyr 438 OH	O02	2.4
ZN	O02	2.5
ZN	O01	2.7

b

ERAP1 atom chain A	DG014 atom chain D	Interatomic distance (Å)
Glu 183 OE1	2X0 1 N10	3.1
Ala 318 N	7GA 2 O	2.7
Glu 320 OE2	2X0 1 N10	3.1
Glu 354 OE1	2X0 1 O12	3.4
Glu 354 OE2	2X0 1 O12	3.1
Glu 376 OE1	2X0 1 O13	2.7
Glu 376 OE2	2X0 1 N10	2.3
Lys 380 NZ	Thr 3 O	3.6
Glu 383 OE1	Phe 4 O	3.9
Glu 383 OE2	Phe 4 O	3.8
Tyr 399 OH	Phe 4 O	4.0
Asp 435 OD2	Thr 3 OG1	2.4
ZN	2X0 1 O12	2.3
ZN	2X0 1 O13	2.2

Supplementary Table 1. ERAP1-inhibitor interactions. a. Intermolecular contacts between DG013 and ERAP1, chain A. b, Intermolecular contacts between DG014 and ERAP1, chain A.

D123	D4
GLU 157	ASN 678
THR 158	MET 726
PHE 159	SER 762
HIS 160	LEU 763
THR 166	SER 792
TYR 167	LEU 793
LYS 170	SER 794
ASP 274	SER 795
LYS 275	THR 796
GLN 278	GLU 797
ILE 311	LYS 798
PRO 312	GLN 800
ASP 313	LYS 828
PHE 314	THR 829
GLN 315	GLN 830
ARG 328	GLU 831
GLU 329	GLN 862
SER 330	LYS 863
LEU 333	PHE 864
ASP 335	GLU 865
GLU 337	LEU 866
SER 339	GLY 867
SER 340	SER 868
SER 343	SER 869
LYS 344	SER 870
LYS 395	HIS 873
GLY 397	MET 876
ASP 406	GLN 904
VAL 410	LEU 905
LEU 413	ARG 906
ASN 414	CYS 907
SER 415	GLN 909
SER 416	GLN 910
HIS 417	THR 914
ALA 427	
GLN 428	
ARG 430	
GLU 431	
PHE 433	
ASP 434	
ASP 435	
GLY 552	

Supplementary Table 2. Interdomain interactions involving residues in domains I,II,III and domain IV that are observed in the closed conformation of ERAP1 (2YD0) and are not present in the open conformation (3MDJ).

10mer peptide	15mer peptide	DG023-L677	DG023-L686	DG23-L838
ARG 168				
	ALA 318			
ILE 347				
		HIS 357	HIS 357	
THR 421	THR 421			
	VAL 436			
SER 553				
ALA 556				
			PHE 644	
GLY 650				
	PHE 674		PHE 674	
				ILE 670
	LEU 677	LEU 677		LEU 677
	ILE 681	ILE 681		
	PRO 682		PRO 682	
			MET 683	
	TYR 684	TYR 684		
	LYS 685	LYS 685		
			LEU 686	
	GLU 688			
SER 729	SER 729			
GLN 730				
	LEU 733	LEU 733		LEU 733
	LEU 734	LEU 734		LEU 734
	VAL 737	VAL 737		VAL 737
	HIS 738			
	GLY 759			
LEU 769	LEU 769	LEU 769		LEU 769
PHE 791				
SER 799 **	SER 799 **			
GLU 802				
PHE 803	PHE 803	PHE 803		PHE 803
	ARG 807	ARG 807		ARG 807
ASP 825				
PRO 833				
GLN 834	GLN 834 **	GLN 834		GLN 834
	THR 837			THR 837
LEU 838				LEU 838
		ARG 841		ARG 841
				GLY 877
			ASN 880	ASN 880
				GLN 881
			ASN 918	
			TRP 921	

Supplementary Table 3. Interdomain interactions between domains I, II, II and domain IV induced by peptide binding for ERAP1-peptide crystal structures and models. Residues in black participate in new interdomain contacts involving ERAP1 residues only. Residues in blue participate in the new interdomain contacts involving both peptide and ERAP1 residues.

ERAP1 Mutation	Primer Sequence
C404S	Forward: 5′ tggagattatttctttggcaaatcttttgacgcaatggaggtag 3′
	Reverse: 5′ ctacctccattgcgtcaaaagatttgccaaagaaataatctcca 3′
C443S	Forward: 5′ gtttcttatgataagggagcttctattctgaatatgctaagggag 3′
	Reverse: 5′ ctcccttagcatattcagaatagaagctcccttatcataagaaac 3′

Supplementary Table 4. Primer sequences used for site directed mutagenesis of ERAP1

a

ERAP1 allele IV, isoform 2

MVFLPLKWSLAIMSFLLSSLLALLTVSTPSWCQSTEASPKRSDGTPFPWNKIRLPEYVIPVHYDLLIHANLTTLTFWGTTKVEITASQPTSTIILHSHHLQISRATLRKGAGERLSEEPLQVLEHPRQ
EQIALLAPEPLLVGLPYTVVIHYAGNLSETFHGFYKSTYRTKEGELRILASTQFEPTAARMAFPCFDEPAFKASFSIKIRREPRHLAISNMPLVKSVTVAEGLIEDHFDVTVKMSTYLVAFIISDFESVS
KITKSGVKVSVYAVPDKINQADYALDAAVTLLEFYEDYFSIPYPLPKQDLAAIPDFQSGAMENWGLTTYRESALLFDAEKSSASSKLDITMTVAHELAHQWFGNLVTMEWWNDLWLNEGFAK
FMEFVSVSVTHPELKVGDYFFGKCFDAMEVDALNSSHPVSTPVENPAQIREMFDDVSYDKGACILNMLREYLSADAFKSGIVQYLQKHSYKNTKNEDLWDSMASICPTDGVKGMDGFCSRS
QHSSSSSHWHQERVDVKTMMNTWTLQRGFPLITITVRGRNVHMKQEHYMKGSDGAPDTGYLWHVPLTFITSKSDMVHRFLLKTKTDVLILPEEVEWIKFNVGMNGYYIVHYEDDGWDSL
TGLLKGTHTAVSSNDRASLINNAFQLVSIGKLSIEKALDLSLYLKHETEIMPVFQGLNELIPMYKLMEKRDMNEVETQFKAFLIRLLRDLIDKQTWTDEGSVSERMLRSELLLLACVHNYQPCVQR
AEGYFRKWKESNGNLSLPVDVTLAVFAVGAQSTEGWDFLYSKYQFSLSSTEKSQIEFALCRTQNKEKLQWLLDESFKGDKIKTQEFPQILTLIGRNPVGYPLAWQFLRKNWNKLVQKFELGSSSI
AHMVMGTTNQFSTRTRLEEVKGFFSSLKENGSQLRCVQQTIETIEENIGWMDKNFDKIRVWLQSEKLEHDPEADATGLERMLESRGPFEQKLISEEDLNMHTEHHHHHH

ERAP1 allele II (K528)

MVFLPLKWSLAIMSFLLSSLLALLTVSTPSWCQSTEASPKRSDGTPFPWNKIRLPEYVIPVHYDLLIHANLTTLTFWGTTKVEITASQPTSTIILHSHHLQISRATLRKGAGERLSEEPLQVLEHPRQ
EQIALLAPEPLLVGLPYTVVIHYAGNLSETFHGFYKSTYRTKEGELRILASTQFEPTAARMAFPCFDEPAFKASFSIKIRREPRHLAISNMPLVKSVTVAEGLIEDHFDVTVKMSTYLVAFIISDFESVS
KITKSGVKVSVYAVPDKINQADYALDAAVTLLEFYEDYFSIPYPLPKQDLAAIPDFQSGAMENWGLTTYRESALLFDAEKSSASSKLGITMTVAHELAHQWFGNLVTMEWWNDLWLNEGFAK
FMEFVSVSVTHPELKVGDYFFGKCFDAMEVDALNSSHPVSTPVENPAQIREMFDDVSYDKGACILNMLREYLSADAFKSGIVQYLQKHSYKNTKNEDLWDSMASICPTDGVKGMDGFCSRS
QHSSSSSHWHQEGVDVKTMMNTWTLQKGFPLITITVRGRNVHMKQEHYMKGSDGAPDTGYLWHVPLTFITSKSDMVHRFLLKTKTDVLILPEEVEWIKFNVGMNGYYIVHYEDDGWDSL
TGLLKGTHTAVSSNDRASLINNAFQLVSIGKLSIEKALDLSLYLKHETEIMPVFQGLNELIPMYKLMEKRDMNEVETQFKAFLIRLLRDLIDKQTWTDEGSVSERMLRSQLLLLACVHNYQPCVQR
AEGYFRKWKESNGNLSLPVDVTLAVFAVGAQSTEGWDFLYSKYQFSLSSTEKSQIEFALCRTQNKEKLQWLLDESFKGDKIKTQEFPQILTLIGRNPVGYPLAWQFLRKNWNKLVQKFELGSSSI
AHMVMGTTNQFSTRTRLEEVKGFFSSLKENGSQLRCVQQTIETIEENIGWMDKNFDKIRVWLQSEKLERMHHHHHH

ERAP1 allele III (R528)

MVFLPLKWSLAIMSFLLSSLLALLTVSTPSWCQSTEASPKRSDGTPFPWNKIRLPEYVIPVHYDLLIHANLTTLTFWGTTKVEITASQPTSTIILHSHHLQISRATLRKGAGERLSEEPLQVLEHPRQ
EQIALLAPEPLLVGLPYTVVIHYAGNLSETFHGFYKSTYRTKEGELRILASTQFEPTAARMAFPCFDEPAFKASFSIKIRREPRHLAISNMPLVKSVTVAEGLIEDHFDVTVKMSTYLVAFIISDFESVS
KITKSGVKVSVYAVPDKINQADYALDAAVTLLEFYEDYFSIPYPLPKQDLAAIPDFQSGAMENWGLTTYRESALLFDAEKSSASSKLGITMTVAHELAHQWFGNLVTMEWWNDLWLNEGFAK
FMEFVSVSVTHPELKVGDYFFGKCFDAMEVDALNSSHPVSTPVENPAQIREMFDDVSYDKGACILNMLREYLSADAFKSGIVQYLQKHSYKNTKNEDLWDSMASICPTDGVKGMDGFCSRS
QHSSSSSHWHQEGVDVKTMMNTWTLQKGFPLITITVRGRNVHMKQEHYMKGSDGAPDTGYLWHVPLTFITSKSDMVHRFLLKTKTDVLILPEEVEWIKFNVGMNGYYIVHYEDDGWDSL
TGLLKGTHTAVSSNDRASLINNAFQLVSIGKLSIEKALDLSLYLKHETEIMPVFQGLNELIPMYKLMEKRDMNEVETQFKAFLIRLLRDLIDKQTWTDEGSVSERMLRSQLLLLACVHNYQPCVQR
AEGYFRKWKESNGNLSLPVDVTLAVFAVGAQSTEGWDFLYSKYQFSLSSTEKSQIEFALCRTQNKEKLQWLLDESFKGDKIKTQEFPQILTLIGRNPVGYPLAWQFLRKNWNKLVQKFELGSSSI
AHMVMGTTNQFSTRTRLEEVKGFFSSLKENGSQLRCVQQTIETIEENIGWMDKNFDKIRVWLQSEKLERMHHHHHH

b

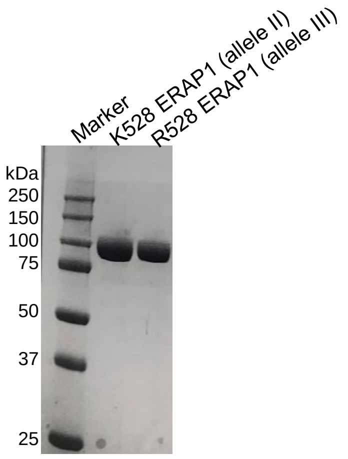

c

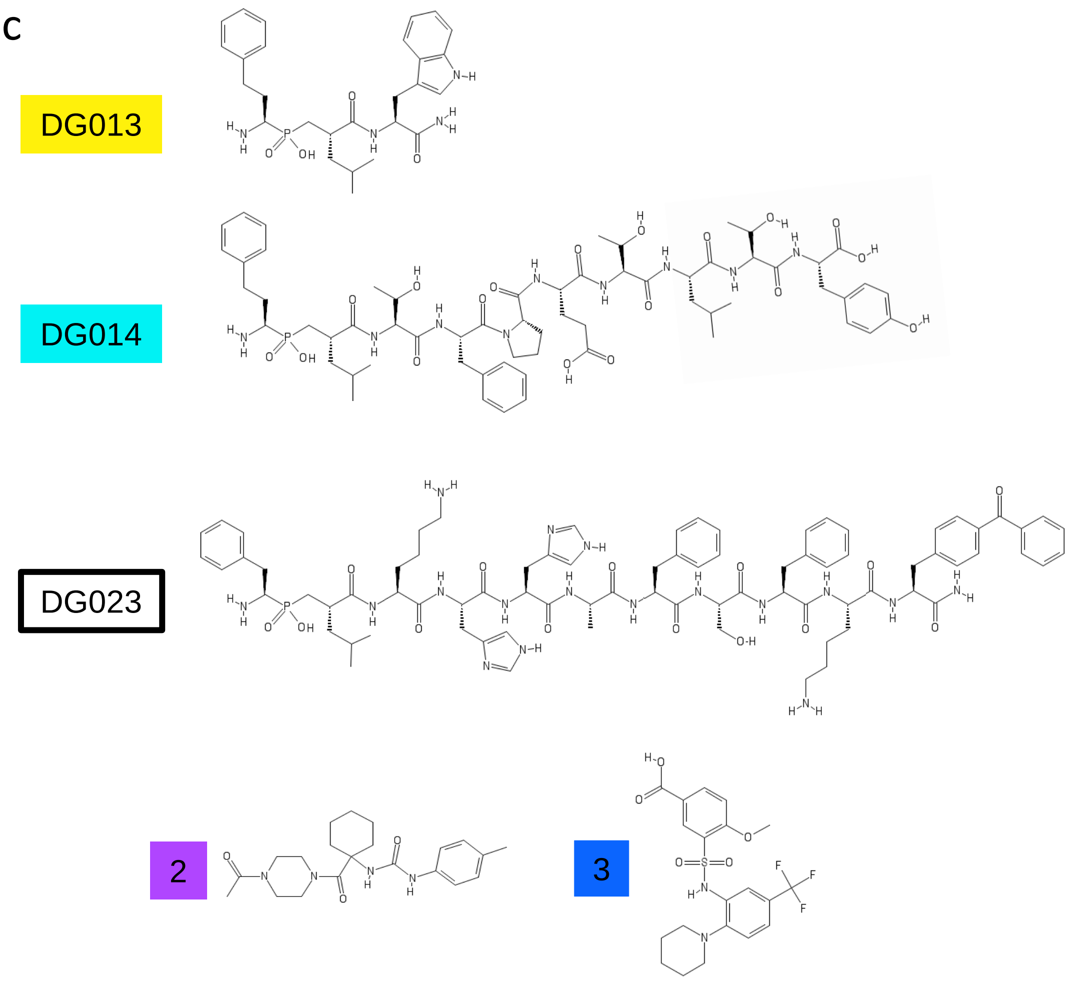

Supplementary Figure 1. **ERAP1 constructs and inhibitors used in this work**. A). Amino acid sequence of ERAP1 constructs used in this work. For crystallization the exon 10 loop (underlined) was replaced with the sequence GGG. B) SDS-PAGE analysis of purified ERAP1 proteins used for SAXS experiments. Coomassie Blue R250 stained 12% acrylamide gel. The purity of the SAXS samples were assessed before and after every SAXS measurement in an independent SDS-PAGE and the samples were observed to be homogenous before and after the measurement. C) Inhibitors and allosteric modulators. Bestatin is a dipeptide analog commonly used as a nonspecific aminopeptidase inhibitor. Leucinethiol is a metalloprotease inhibitor that reacts with active site metal ion. The octamer peptide SIINFEKL is a model T cell epitope and has been characterized as an ERAP1 substrate. DG013 and DG014 and DG023 are peptidomimetic inhibitors (trimer, decamer, and unamer respectively) where the first peptide bond is replaced by a nonhydrolyzable tetragonal transition-state mimic phosphinic group. DG023 also has an unnatural photocrosslinker amino acid (L-4-benzoyl-phenylalanine) as its C-terminus. Compounds **2** and **3** were previously characterized as a highly specific inhibitors of ERAP1-catalyzed peptide hydrolysis.

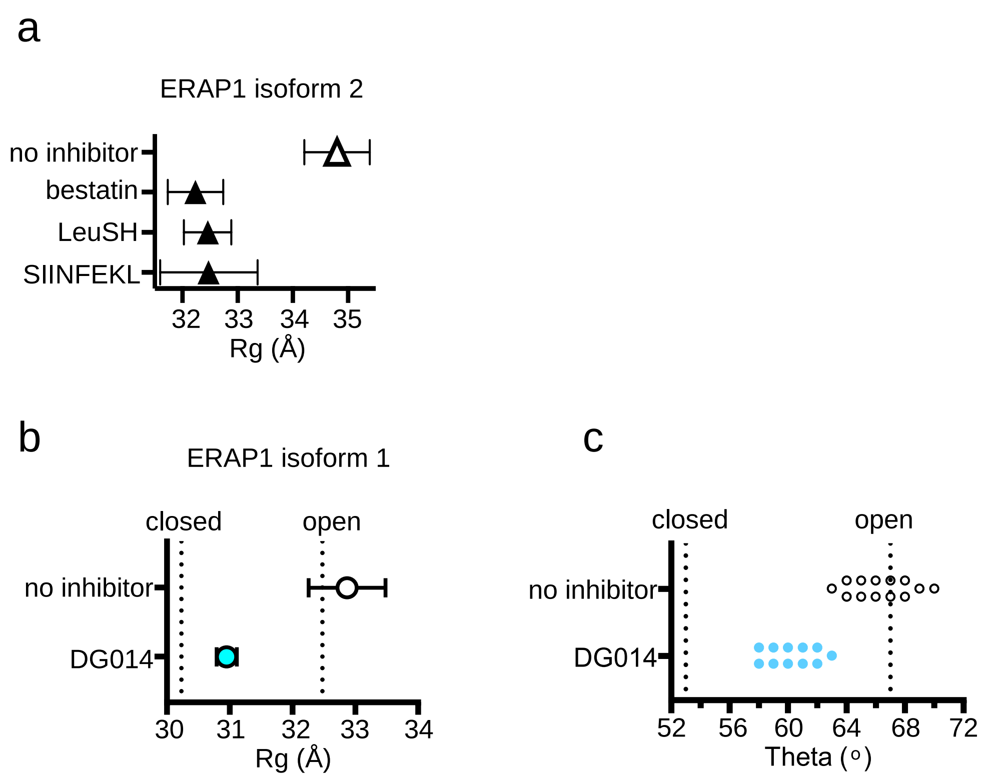

Supplementary Figure 2. Substrates and inhibitors induce closed ERAP1 conformation. a, R_g determined by Guinier analysis decreases in the presence of saturating concentrations of inhibitor or substrate, indicating that ERAP1 adopts a more compact structure in these conditions. Error bars represent uncertainties for the calculated R_g value (slope of log intensity vs. q) and each data point correspond to an independent experiment with a single sample (n=1). b and c, ERAP1 adopts closed conformation in presence of substrate-mimic inhibitor DG014 as measured by (b) R_g analysis and (c) theta calculations done by fitting into MD models. Error bars in (b) represent uncertainties for the calculated R_g value and data represents one independent experiment with n=1 in the presence and absence of the inhibitor.

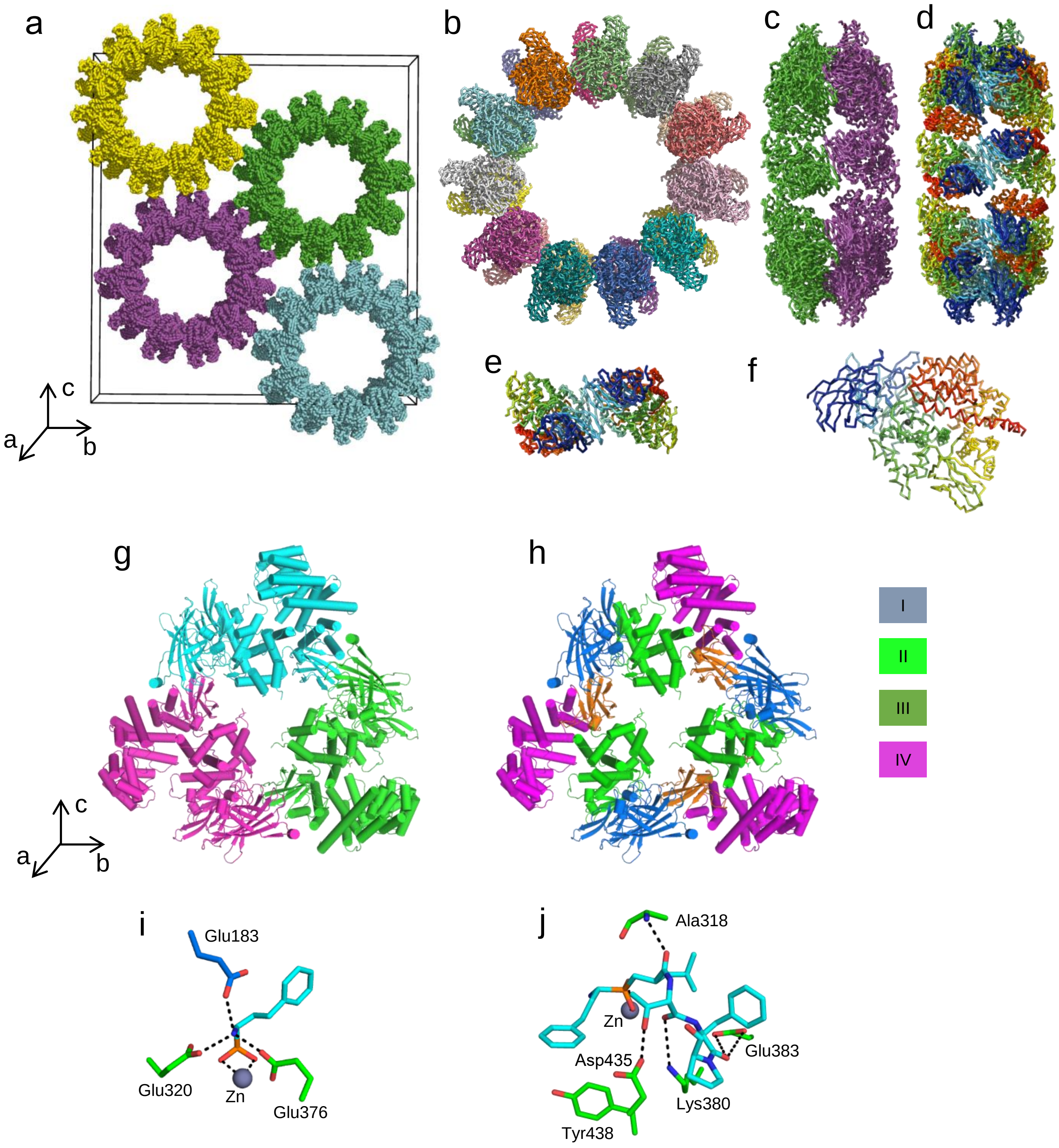

Supplementary Figure 3. **ERAP1 crystal structure overviews**. a-f, ERAP1 (Δ exon 10 loop):DG013 closed crystal structure overview. a, Four ASU per unit cell. b, Ribbon view of 22 ERAP1 monomer in ASU colored by chain. c, rotated 90 degrees relative to (b), ASU consists of two stacked 11mer rings, here each 11mer ring colored green and magenta, respectively. d, As in (C) colored as chainbow. e, As in (d) showing two monomers. f, ERAP1 monomers are in closed conformation, one chain shown as chainbow. g-j, ERAP1:DG014 open crystal structure overview. g, Asymmetric unit has three ERAP1 monomers, colored by chain. h, View as in (A) colored by domain. i, DG014 N-terminal homophenylalanine is bonded to active site residues and catalytic zinc. j, Interatomic contacts between DG014 and ERAP1.

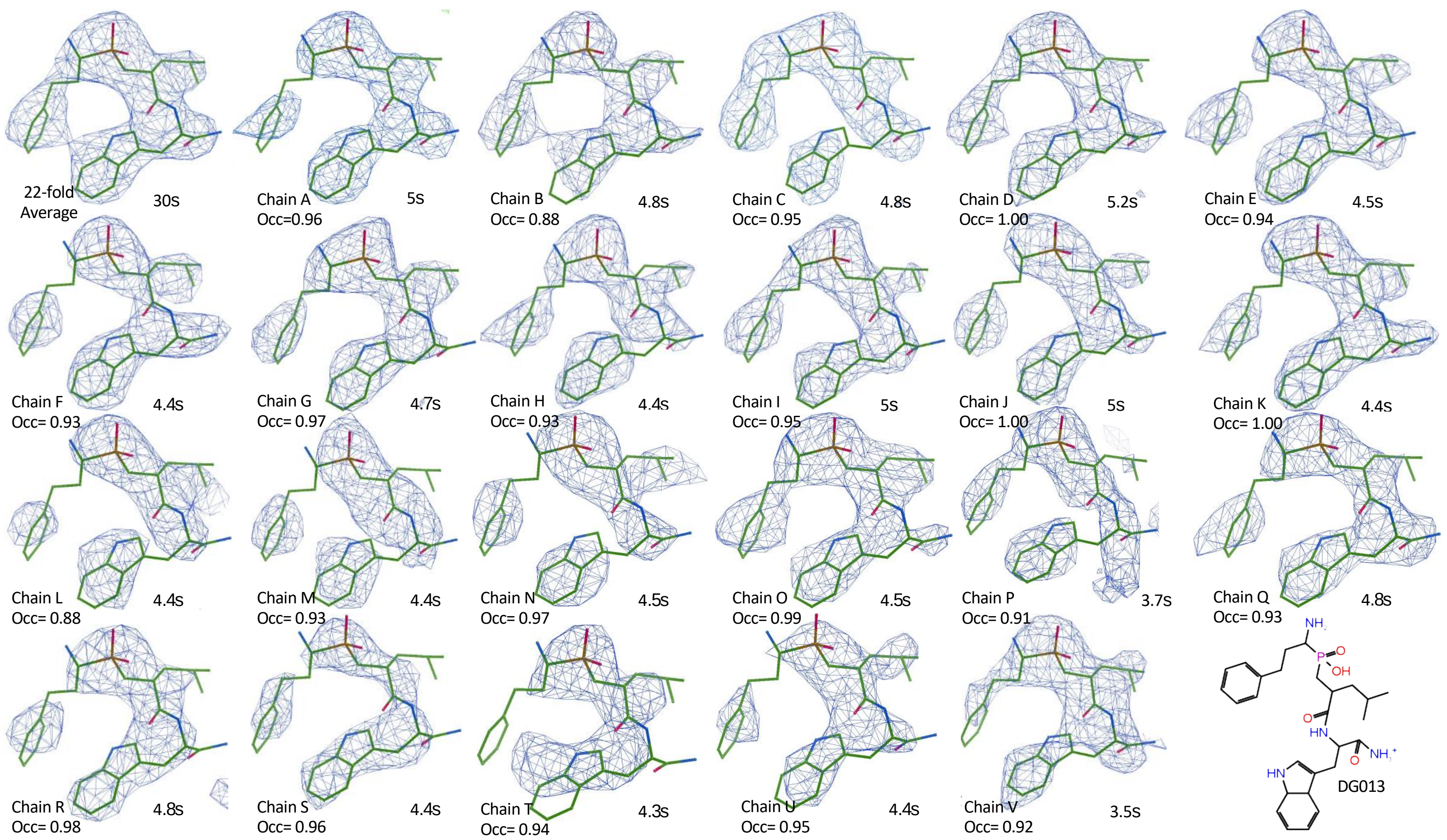

Supplementary Figure 4. **DG013 ligand omit-map electron density** . Map sections shown in the vicinity of DG013 in the 22-fold averaged omit map (upper left) and in the unaveraged omit map for each of the 22 molecules in the asymmetric unit. All atoms shown were omitted from the map calculation, and no map trimming was performed. Refined occupancy for DG013 and the sigma level of the map are shown at the bottom of each panel. Bottom right, chemical structure of DG013.

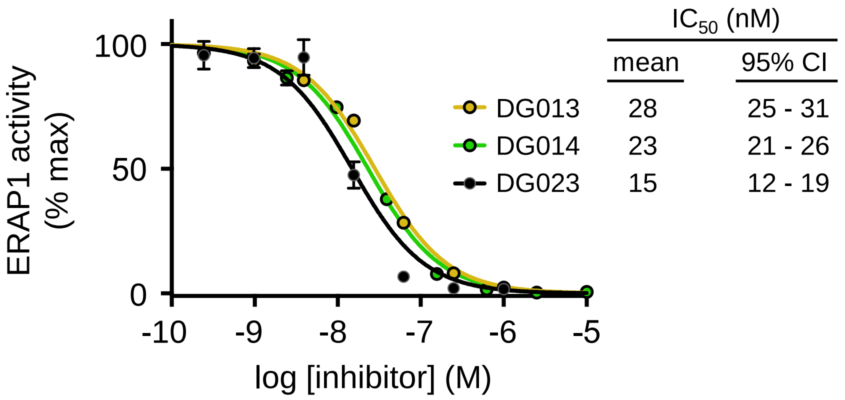

Supplementary Figure 5. **Substrate analogs inhibit ERAP1 hydrolysis of leucine-AMC.** Shown are normalized activity curves for DG013, DG014, and DG023. Representative data from one of two experiments.

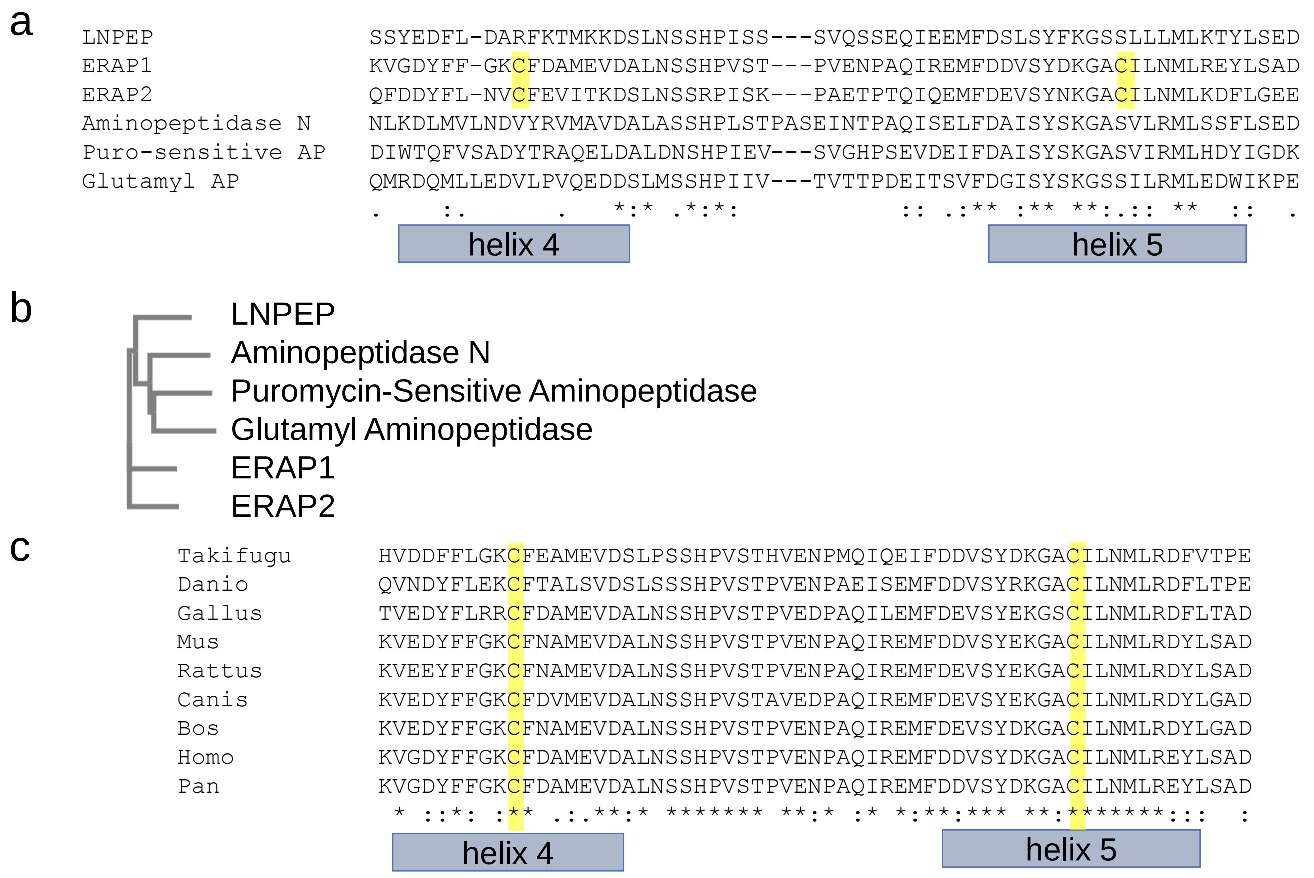

Supplementary Figure 6. **Sequence alignment of ERAP1 orthologs and paralogs**. A, ERAP1 and ERAP2 alone among M1 family zinc aminopeptidases have a disulfide bond linking H4 and H5. Sequence alignment of domain II of M1 zinc aminopeptidases in *Homo sapiens*. B, Phylogram of M1 zinc aminopeptidases in *Homo sapiens.* C, ERAP1 H4-H5 disulfide is evolutionarily conserved. Sequence alignment of domain II of ERAP1 from selected model organisms. All alignments were performed using Clustal Omega and UniprotKB protein sequence designations.

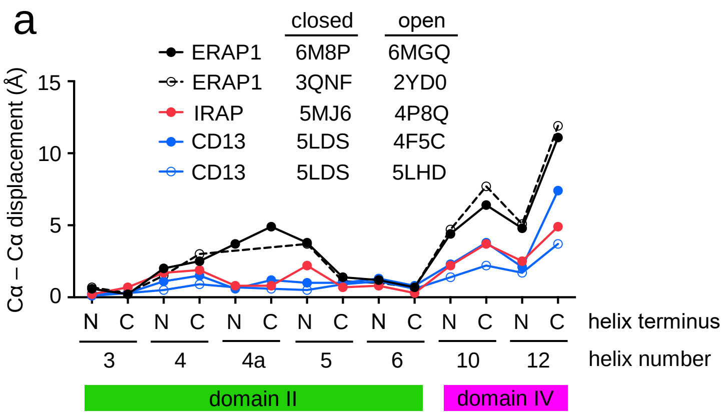

Supplementary Figure 7. **Coupling of active site rearrangements with interdomain interactions.** a) Displacement between open and closed crystal structures for the three M1 family aminopeptidases, indicated as the difference in Ca-Ca distance for N- and C-terminal residues of helices near the domain interface. For PDBs with multiple chains, chain A was used.

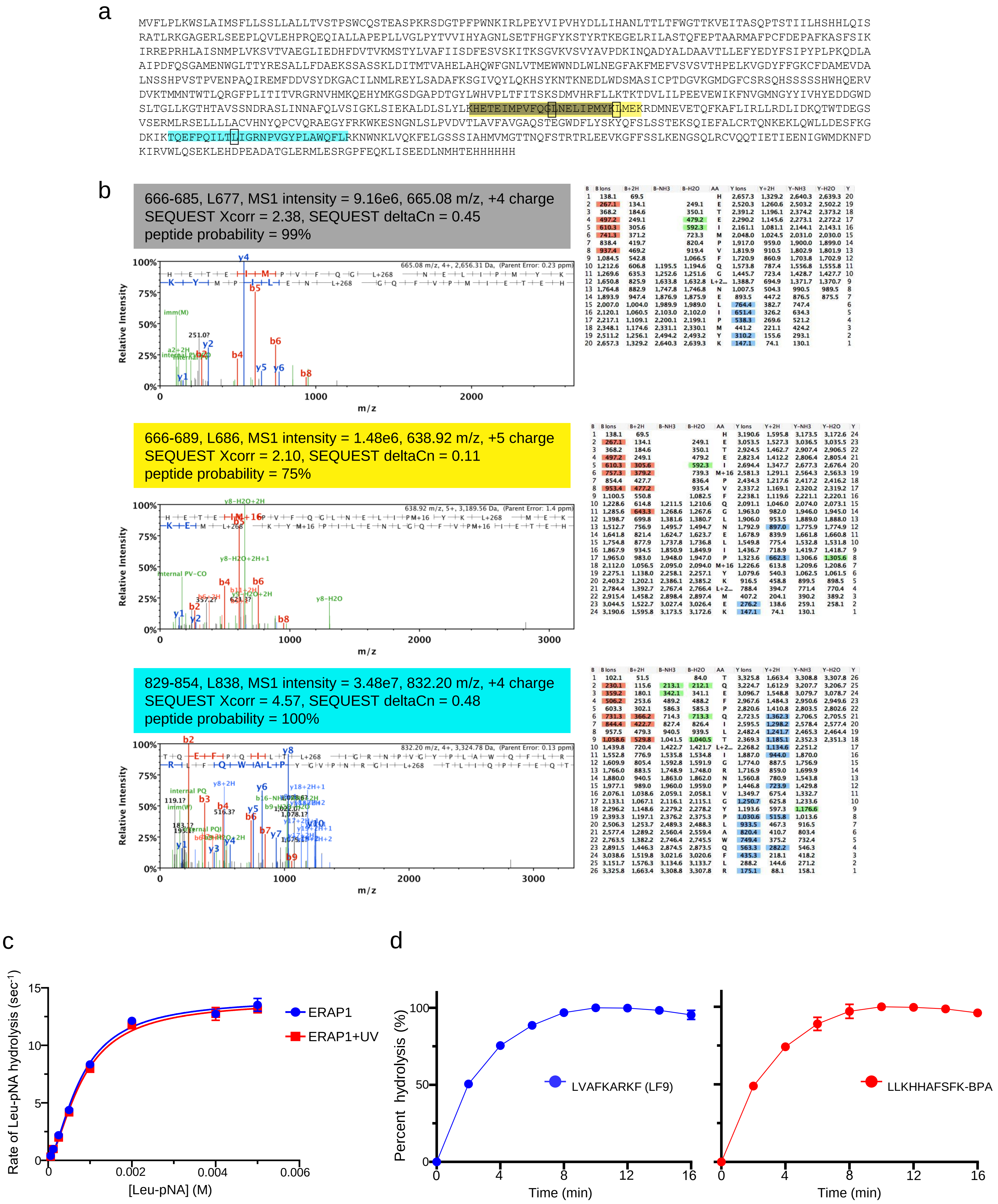

Supplementary Figure 8. **MS/MS data for ERAP1 tryptic peptides modified by photoactivatable crosslinker and control crosslinking experiments**. a, Identified peptide sequences within ERAP1 are highlighted gray, yellow, or cyan. Crosslinked residues are boxed. b, MS/MS spectra and fragmentation tables for crosslinked ERAP1 peptides generated from Scaffold4, colored as in (a). c, UV-irradiation as used for crosslinking experiment does not alter ERAP1 catalytic capacity, as assayed by Leu-AMC hydrolysis. d, C-terminal BPA group used for crosslinking experiment does not interfere with processing by ERAP1. Removal of peptide N-terminal Leu by ERAP1 was followed by a coupled enzyme assay. Activity assays in (c) and (d) were performed in two independent experiments (n=2) and error bars represent the variation between the replicates in an experiment.

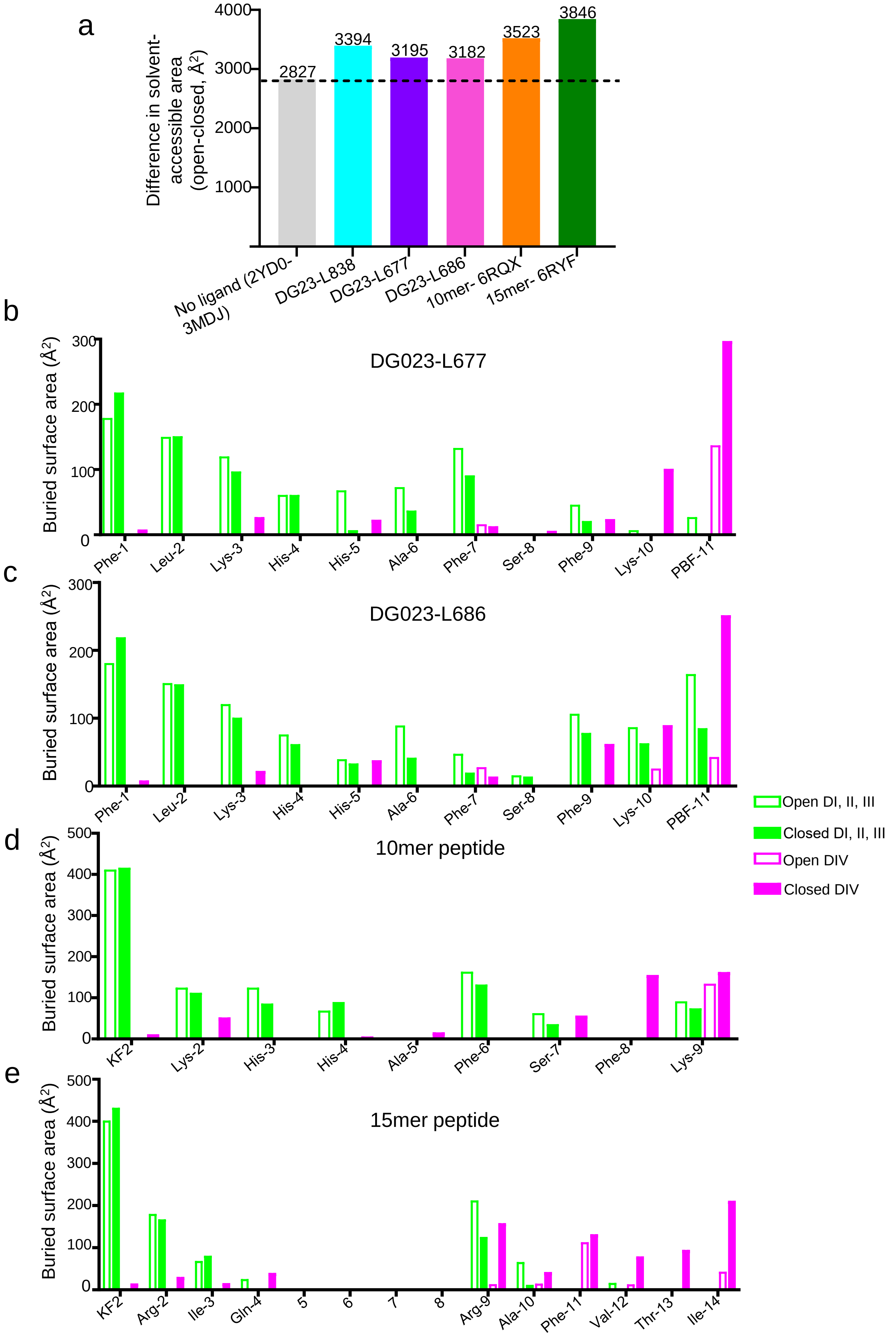

Supplementary Figure 9. **Solvent-accessible and buried surface area analysis of ERAP1 without and with long peptide ligand.** a, The amount of ERAP1 surface area buried by domain closure, i.e., the solvent-accessible area of the open conformer minus that of the closed conformer, is larger in the presence of bound peptide. 2YD0 (closed) and 3MDJ (open) were used as no ligand controls because of the absence of long peptides in the structures. DG023 peptide models (L677 and L686) and crystal structures of ERAP1 bound with 10mer and 15mer were used to calculate the solvent-accessible surface area (SAA) of open and closed conformers using PISA. b-e, Buried surface area of peptide residues in domain I, II, III (green) and domain IV (magenta) in open (open bars) and closed conformer (closed bars) along the length of the peptide.

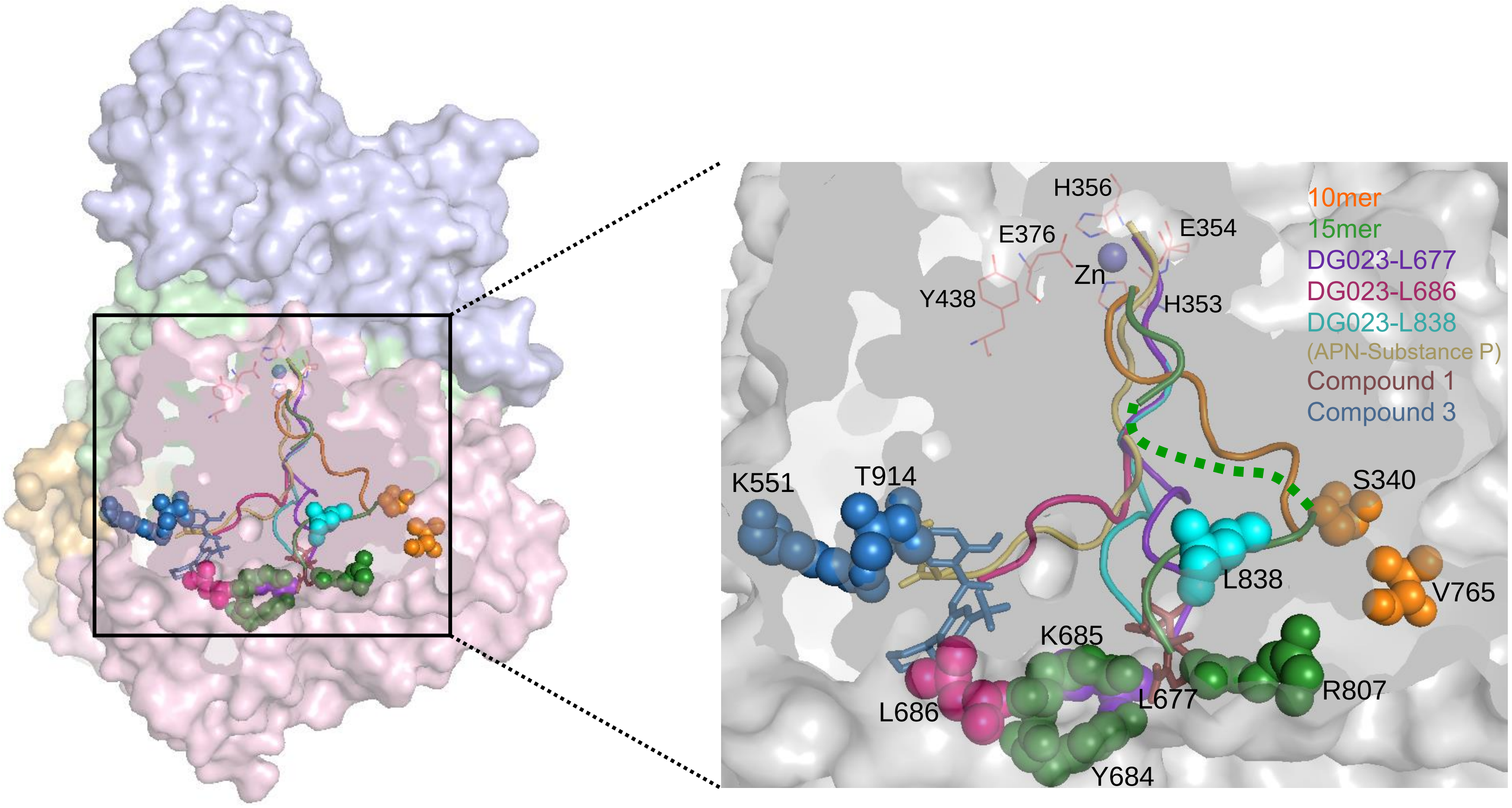

Supplementary Figure 10. **Peptide C-terminal and allosteric activator binding sites.** *Left,* cutaway view of ERAP1 with surface colored by domain. *Right,* close-up of view of substrate binding cavity with ERAP1 surface colored grey . ERAP1 residues in contact with (within 3.5Å) the C-terminal residues of co-crystalized 10mer peptide (orange) or 15mer peptide (6RQX, 6RYF), ERAP1 residues identified in this study using an 11-mer peptide with C-terminal photo-crosslinking residue (L677-DG023, violet; L686-DG023, magenta; L838-DG023, cyan), small-molecule allosteric activator compound 1 co-crystallized with ERAP1 (compound 1, maroon, 6T6R), a small-molecule allosteric activator compound 3 docked onto ERAP1 (teal), and corresponding residues from the ERAP1 homolog human aminopeptidase N (APN) in contact with the C-terminal residue of co-crystalized 10mer substance P peptide (yellow, PDBID: 4HOM, PMID 23071329), are indicated with spheres color-coded to match the peptides and compounds. Contact residues for compound 1 are shared with 15mer peptide and colored green. Active site Zn, coordinating residues, and catalytic Tyr438 are shown at top.

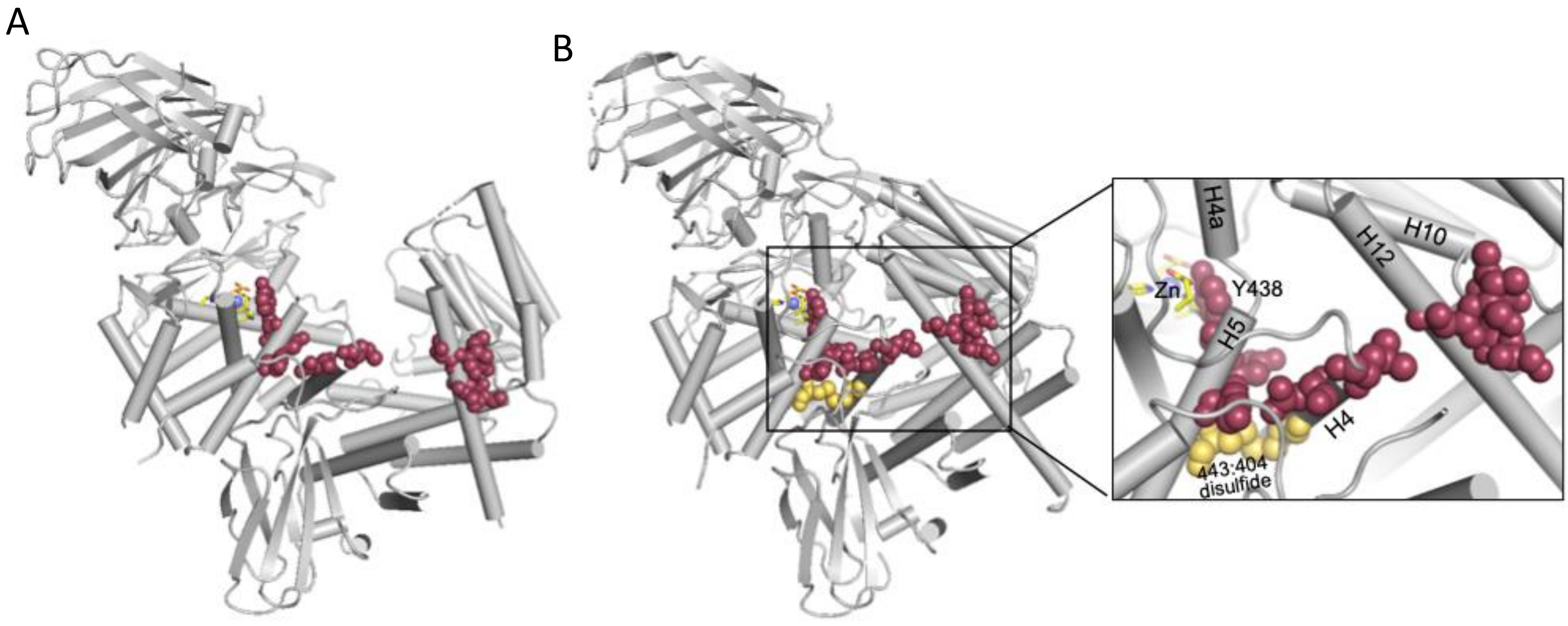

Supplementary Figure 11. **ERAP1 domain motion and a network of correlated motions**. Open (A) and closed (B) conformations of ERAP1 (6RYF), with red spheres showing residues along a path of correlated motions identified by analysis of molecular dynamics simulation. The path connects peptide C-terminal binding sites and active site residue Y438 in the closed conformation but is disconnected in the open conformation. *Inset* shows the correlated path along with the C404-C443 disulfide connecting helices 4 and 5 near the interface with helices 10 and 12.

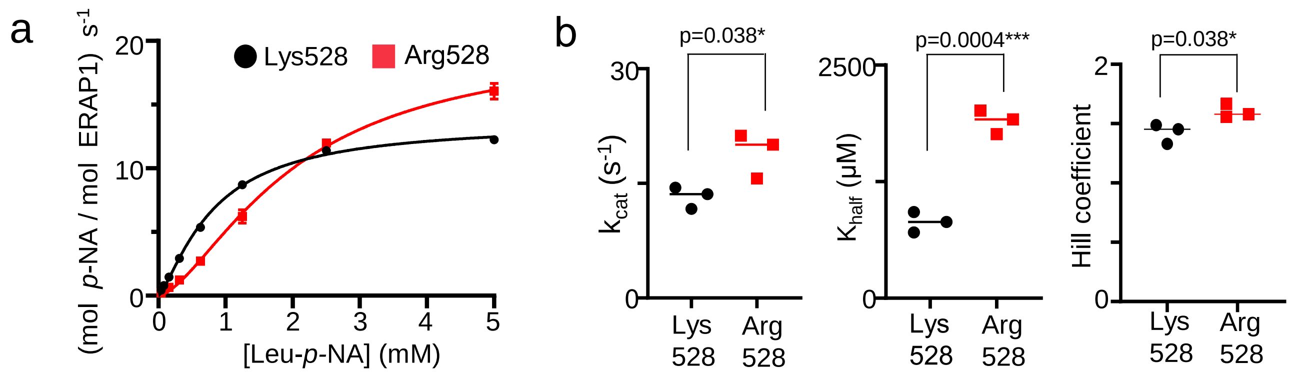

Supplementary Figure 12. **Disease-associated polymorphism at position 528 modulates ERAP1 enzymatic activity**. a, Rate of L-pNA hydrolysis by Lys528 and Arg528 were globally fit to an allosteric sigmoidal curve. Error bars represent the variation in replicates in an independent experiment. b, Fit parameters from three individual experiments (n=2). Asterisks indicate statistical significance derived from unpaired two-tailed t-test along with the calculated p values.
