# Supplementary material for: Conformational dynamics linked to domain closure and substrate binding explain the ERAP1 allosteric regulation mechanism
Source: Nat Commun. 2021 Sep 6;12:5302. doi: 10.1038/s41467-021-25564-w (PMC8421391; doi:10.1038/s41467-021-25564-w)
Supplement: Supplementary file 2 — Reporting Summary [file 41467_2021_25564_MOESM2_ESM.pdf]

nature research

Corresponding author(s): Lawrence Stern

Last updated by author(s): Jul 27, 2021

Reporting Summary

Nature Research wishes to improve the reproducibility of the work that we publish. This form provides structure for consistency and transparency in reporting. For further information on Nature Research policies, see our Editorial Policies and the Editorial Policy Checklist.

Statistics

For all statistical analyses, confirm that the following items are present in the figure legend, table legend, main text, or Methods section.

n/a	Confirmed	
☐	☒	The exact sample size (n) for each experimental group/condition, given as a discrete number and unit of measurement
☐	☒	A statement on whether measurements were taken from distinct samples or whether the same sample was measured repeatedly
☐	☒	The statistical test(s) used AND whether they are one- or two-sided<br>*Only common tests should be described solely by name; describe more complex techniques in the Methods section.*
☒	☐	A description of all covariates tested
☒	☐	A description of any assumptions or corrections, such as tests of normality and adjustment for multiple comparisons
☐	☒	A full description of the statistical parameters including central tendency (e.g. means) or other basic estimates (e.g. regression coefficient) AND variation (e.g. standard deviation) or associated estimates of uncertainty (e.g. confidence intervals)
☐	☒	For null hypothesis testing, the test statistic (e.g. F, t, r) with confidence intervals, effect sizes, degrees of freedom and P value noted<br>*Give P values as exact values whenever suitable.*
☒	☐	For Bayesian analysis, information on the choice of priors and Markov chain Monte Carlo settings
☒	☐	For hierarchical and complex designs, identification of the appropriate level for tests and full reporting of outcomes
☒	☐	Estimates of effect sizes (e.g. Cohen's d, Pearson's r), indicating how they were calculated

Our web collection on statistics for biologists contains articles on many of the points above.

Software and code

Policy information about availability of computer code

Data collection

ATSAS v2.5.2 (SAXS)
SCÅTTER v3.0g (SAXS)
XDS version June 1 2017 (crsytallography)
STARANISO v2.0.06 (crystallography)
ProtoemeDiscover v2.1.1.21 (mass spectrometry)

Data analysis

AutoRG v3.0(SAXS)
FOXS (SAXS)
Phenix v1.19.2-4158 (Crystallographic refinement), including Polder (Map calculation), Phaser v2.7.16, eLBOW, MolProbity v4.02
CCP4 v7.1 (Crystallographic analysis and refinement), including Zanuda
Coot v0.9.5 (model building)
Prodrg 2(model building)
Allosmod main.e7ad349 (model building)
Scaffold v4 and v5.0.1 (mass spectra analysis)
Pymol v2.3.4 (visualization)
PDB2PQR v2.1.1 (electrostatics)
DelPhi v8.0(electrostatics)
PDBePISA v1.52 (protein-protein interaction analysis)
GraphPad Prism v7.0 and 8.0 (graphing and statistical analysis)

For manuscripts utilizing custom algorithms or software that are central to the research but not yet described in published literature, software must be made available to editors and reviewers. We strongly encourage code deposition in a community repository (e.g. GitHub). See the Nature Research guidelines for submitting code & software for further information.

Data

Policy information about availability of data

All manuscripts must include a data availability statement. This statement should provide the following information, where applicable:
- Accession codes, unique identifiers, or web links for publicly available datasets
- A list of figures that have associated raw data
- A description of any restrictions on data availability

Crystallographic diffraction data and atomic coordinates are available in the PDB (ID:6MGQ and ID:6M8P) and raw datasets are available in the SBGRID repository (#605 and 606)

Field-specific reporting

Please select the one below that is the best fit for your research. If you are not sure, read the appropriate sections before making your selection.

☒ Life sciences ☐ Behavioural & social sciences ☐ Ecological, evolutionary & environmental sciences

For a reference copy of the document with all sections, see nature.com/documents/nr-reporting-summary-flat.pdf

Life sciences study design

All studies must disclose on these points even when the disclosure is negative.

Sample size	All activity assays and binding studies experiments had at least two replicate samples. Sample size was chosen based on the low variations observed between samples in similar experiments. Sample to sample variation was low in all biochemical assays.
Data exclusions	Crystallographic reflections beyond a ellipsoidal resolution limit determined by CC1/2 estimation were excluded. In crosslinking experiment, one peptide identified with m/z consistent with a +268 modification of an ERAP1 tryptic peptide was excluded from analysis as it appeared in the dataset generated in the absence of DG023 or BPA.
Replication	Biochemical assays were performed in at least two individual experiments, with at least two replicate samples included for each data point in each experiment.
Randomization	No randomization protocols were used for activity assays or binding studies because individual samples were assessed in parallel and not sequentially. For SAXS, samples were assessed sequentially because randomization was irrelevant here.
Blinding	Activity assays, binding studies and some scattering data collection were not blinded because investigator's bias would not affect the data collection or analysis. Crystallographic data collection and some SAXS data collection was blinded as it was collected by beamline staff.

Reporting for specific materials, systems and methods

We require information from authors about some types of materials, experimental systems and methods used in many studies. Here, indicate whether each material, system or method listed is relevant to your study. If you are not sure if a list item applies to your research, read the appropriate section before selecting a response.

Materials & experimental systems

n/a	Involved in the study	
☒	☐	Antibodies
☐	☒	Eukaryotic cell lines
☒	☐	Palaeontology and archaeology
☒	☐	Animals and other organisms
☒	☐	Human research participants
☒	☐	Clinical data
☒	☐	Dual use research of concern

Methods

n/a	Involved in the study	
☒	☐	ChIP-seq
☒	☐	Flow cytometry
☒	☐	MRI-based neuroimaging

Eukaryotic cell lines

Policy information about cell lines

Cell line source(s)	Sf9 (CRL-1711) and HiFive (BTI-Tn-5B1-4, CRL-10859) insect cell lines were obtained from ATCC
Authentication	Cell lines used were not authenticated
Mycoplasma contamination	Cell lines used were not tested for mycoplasma contamination

Commonly misidentified lines (See ICLAC register)	No commonly misidentified cell lines were used in the study
